# Quantitative assessment of plant-arthropod interactions in forest canopies: A plot-based approach

**DOI:** 10.1371/journal.pone.0222119

**Published:** 2019-10-23

**Authors:** Martin Volf, Petr Klimeš, Greg P. A. Lamarre, Conor M. Redmond, Carlo L. Seifert, Tomokazu Abe, John Auga, Kristina Anderson-Teixeira, Yves Basset, Saul Beckett, Philip T. Butterill, Pavel Drozd, Erika Gonzalez-Akre, Ondřej Kaman, Naoto Kamata, Benita Laird-Hopkins, Martin Libra, Markus Manumbor, Scott E. Miller, Kenneth Molem, Ondřej Mottl, Masashi Murakami, Tatsuro Nakaji, Nichola S. Plowman, Petr Pyszko, Martin Šigut, Jan Šipoš, Robert Tropek, George D. Weiblen, Vojtech Novotny

**Affiliations:** 1 Biology Centre of the Czech Academy of Sciences, Ceske Budejovice, Czech Republic; 2 German Centre for Integrative Biodiversity Research (iDiv) Halle-Jena-Leipzig, Leipzig, Germany; 3 Faculty of Science, University of South Bohemia, Ceske Budejovice, Czech Republic; 4 Faculty of Science, Chiba University, Chiba, Japan; 5 New Guinea Binatang Research Center, Madang, Papua New Guinea; 6 Conservation Ecology Center, Smithsonian Conservation Biology Institute; Front Royal, VA, United States of America; 7 ForestGEO, Smithsonian Tropical Research Institute, Panama City, Panama; 8 Maestria de Entomologia, Universidad de Panama, Panama City, Panama; 9 Faculty of Science, University of Ostrava, Ostrava, Czech Republic; 10 Graduate School of Agricultural and Life Sciences, The University of Tokyo, Furano, Japan; 11 School of Biological Sciences, University of Bristol, Bristol, United Kingdom; 12 National Museum of Natural History, Smithsonian Institution, Washington, DC, United States of America; 13 Tomakomai Experimental Forest, Hokkaido University, Tomakomai, Japan; 14 Institute of Botany, Czech Academy of Sciences, Brno, Czech Republic; 15 Department of Zoology, Fisheries, Hydrobiology and Apiculture, Mendel University in Brno, Brno, Czech Republic; 16 Department of Ecology, Faculty of Science, Charles University, Prague, Czech Republic; 17 Bell Museum and Department of Plant & Microbial Biology, University of Minnesota, Saint Paul, MN, United States of America; Helmholtz Centre for Environmental Research - UFZ, GERMANY

## Abstract

Research on canopy arthropods has progressed from species inventories to the study of their interactions and networks, enhancing our understanding of how hyper-diverse communities are maintained. Previous studies often focused on sampling individual tree species, individual trees or their parts. We argue that such selective sampling is not ideal when analyzing interaction network structure, and may lead to erroneous conclusions. We developed practical and reproducible sampling guidelines for the plot-based analysis of arthropod interaction networks in forest canopies. Our sampling protocol focused on insect herbivores (leaf-chewing insect larvae, miners and gallers) and non-flying invertebrate predators (spiders and ants). We quantitatively sampled the focal arthropods from felled trees, or from trees accessed by canopy cranes or cherry pickers in 53 0.1 ha forest plots in five biogeographic regions, comprising 6,280 trees in total. All three methods required a similar sampling effort and provided good foliage accessibility. Furthermore, we compared interaction networks derived from plot-based data to interaction networks derived from simulated non-plot-based data focusing either on common tree species or a representative selection of tree families. All types of non-plot-based data showed highly biased network structure towards higher connectance, higher web asymmetry, and higher nestedness temperature when compared with plot-based data. Furthermore, some types of non-plot-based data showed biased diversity of the associated herbivore species and specificity of their interactions. Plot-based sampling thus appears to be the most rigorous approach for reconstructing realistic, quantitative plant-arthropod interaction networks that are comparable across sites and regions. Studies of plant interactions have greatly benefited from a plot-based approach and we argue that studies of arthropod interactions would benefit in the same way. We conclude that plot-based studies on canopy arthropods would yield important insights into the processes of interaction network assembly and dynamics, which could be maximised via a coordinated network of plot-based study sites.

## Introduction

Forest canopies represent one of the most diverse environments on the planet [1], harbouring a large proportion of terrestrial arthropod diversity estimated at 6.8 million species [2]. At the same time, canopies are among the least explored habitats due to the logistical challenges of accessibility [1]. This combination of high diversity and inaccessibility has fascinated biologists for more than 150 years [1].

The development of single-rope climbing and fogging has provided ecologists with efficient tools for researching canopy arthropod communities, generating several influential studies e.g. [3, 4, 5]. Such studies spurred the development of new methods of access that nowadays include canopy walkways, canopy rafts, balloons, cherry pickers, or canopy cranes [1]. Canopy studies have contributed to our understanding of species global diversity and biotic interactions [2, 6], but, as pointed out by Lowman et al. [1], *“…the real challenge is ahead*. *Canopy organisms*, *both mobile and sessile*, *must be surveyed and their roles measured*.*”*

Research into canopy arthropods has progressed from species inventories to the study of their interactions, allowing us to understand how hyper-diverse communities of canopy arthropods are maintained [7]. Particular sampling methods are suitable for different systems and questions concerning the various roles arthropods play in forest canopies [1] (Table 1). Methods that allow access to individual branches or certain parts of the canopy are suitable for exploratory studies on arthropod diversity, detailed surveys focused on specific taxa, or manipulative experiments e.g. [8, 9]. They also allow for comparative studies across various canopy microhabitats and their arthropod communities [10]. However, to fully census interactions between arthropods and plants on the level of the canopy as a whole, sampling methods must provide access to the entire canopy, from the terminal branches, through the inner canopy, to the lower branches. This is because arthropod species composition may differ considerably among various parts of the canopy [11], reflecting variation in resource availability and leaf traits [12]. Neglecting some parts of the canopy, therefore, has the potential to influence the results of the census. In addition, methods suitable for censusing canopy arthropod interactions must facilitate the sampling of arthropods in such a way that enables the reliable reconstruction of the interaction network. In the tropics, transient herbivorous arthropod species (i.e. species with no lasting association to the sampled plant) can comprise up to 20% of species found on a particular tree [13]. Thus, dead arthropods sampled from a plant do not constitute reliable interactions. To reliably reconstruct interaction networks, one needs to either sample live arthropods for feeding trials [14] or a use molecular detection of trophic interactions [15]. Similarly, it is necessary to map ant nests rather than simply sample individual ants, as up to half of the ants foraging in a tree are tourists from surrounding vegetation [16].

**Table 1:** Summary characteristics of forest canopy sampling methods that allow active sampling of arthropods by manual search, beating, sweeping, or fogging. The trapping methods are not listed. Characteristics include Canopy accessibility (accessibility of tree strata: T (terminal branches), U (upper canopy), L (lower canopy), I (inner canopy)); suitable Scale of sampling (whole canopy vs. individual branches), Arthropod taxa sampled (E (endophytic), T (trunk-nesting), N (non-flying exophytic herbivores and predators), F (flying).

Method	Canopy accessibility	Scale	Arthropod taxa	Team size	Costs	Replicability	Site availability	References
Canopy crane	T,U,L	Whole canopy, branches	E,N,F	Medium	High	Low	Low	Basset et al. [17]; Ødegaard [18]; Wardhaugh [19]
Cherry picker	T,U,L	Whole canopy, branches	E,N,F	Medium	High	High	Medium	Corff and Marquis [20]; Volf et al. [21]
Felling	T,U,L,I	Whole canopy	E,T,N	Large	Medium	High	Medium	Whitfeld et al. [22]; Redmond et al. [23]
Canopy rafts	T,U	Branches	E,N,F	Medium	High	Low	High	Lowman et al.[8]
Canopy walks	U,L,I	Branches	E,N,F	Medium	Medium	Low	Low	Reynolds and Crossley [24]
Fogging	T,U,L,I	Whole canopy	N*,F*	Small	Low	High	High	Erwin [3]; Kitching et al. [25]
Tree climbing	U,L,I	Branches	E,T,N,F	Small	Low	High	High	Lowman [26]; Schowalter and Zhang [27]

* indicates that dead insects are sampled); minimal required Team size; relative operational Costs; Replicability (ease and practicality of replication); Site availability (low—limited sites with crane or walkway access; medium–available access road for cherry picker, felling not permissible in protected forests and other situations; high—almost all forests can be sampled); and key References.

Most importantly, for a quantitative analysis of arthropod interaction networks, the methods should allow structured sampling across large parts of the canopy, thus including all species in proportion to their abundance [7, 21, 28]. Previous studies often focused on sampling individual tree species, individual trees or selected constituent parts. Selective sampling is particularly beneficial for exploring insect-plant interactions in a phylogenetical or evolutionary framework as it allows the researcher to focus on particular lineages of interest [29, 30]. Methods that employ selective sampling are also valuable when assessing herbivore specialization or the effects of host-plant traits on insect community structure. This is because all focal species can be sampled with equal effort, thus allowing for direct comparisons between herbivore or host species [14, 31]. However, a drawback of selective sampling is that it does not facilitate quantitative network structure analyses, because it tends to skew interaction frequencies, over- or underestimate specialization and diversity, and biases network structure [7]. In particular, it typically omits a high proportion of the arthropod and plant taxa co-existing at the sites, hence not reflecting species diversity and network structure at the whole forest level. We argue that for interaction network analyses, a plot-based approach, where entire plots are censused for plants and arthropods, is preferable, as it more accurately reflects the diversity and abundance of the available resources [21, 23].

**Fig 1:**
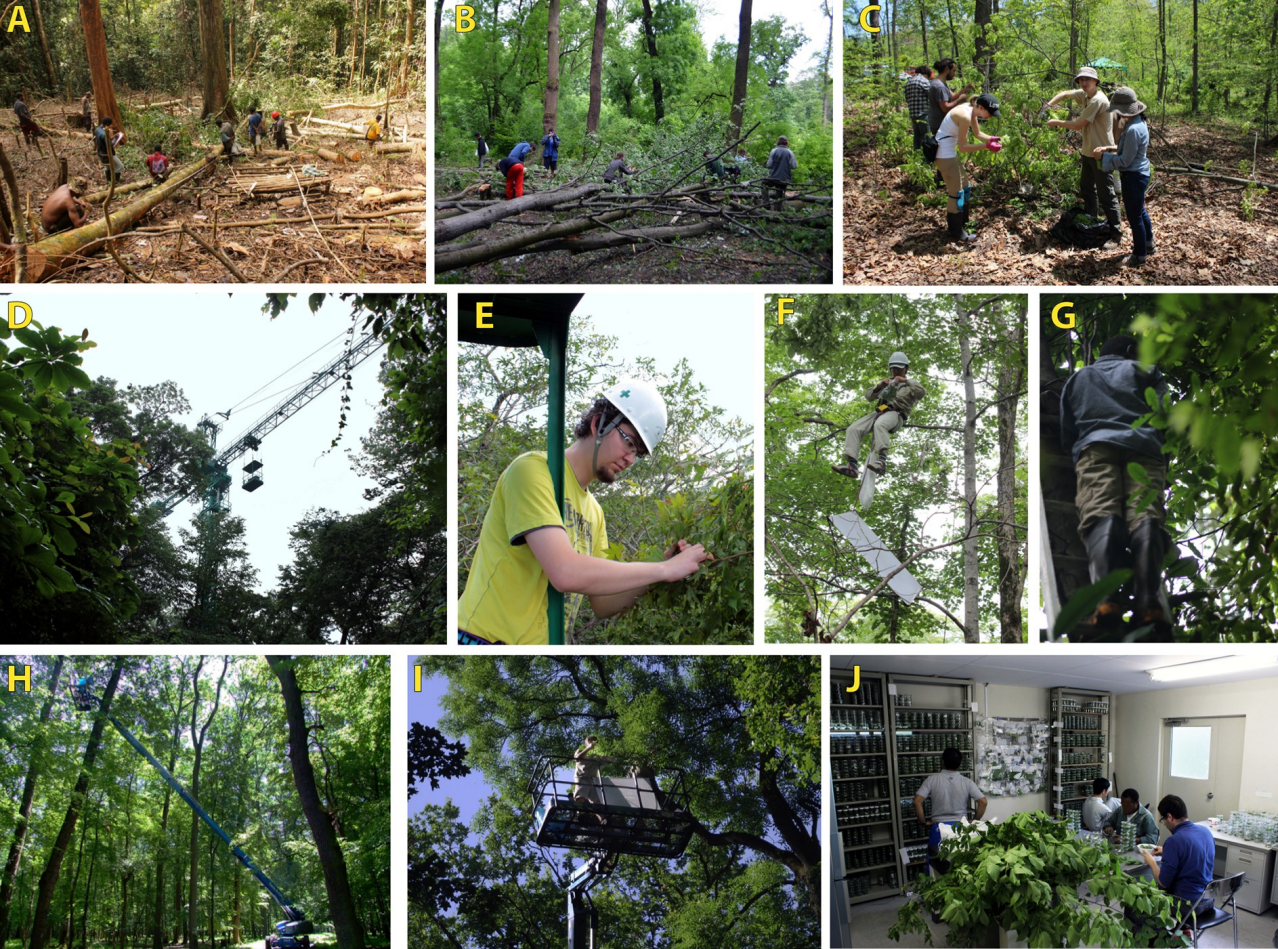
Photos from the field. Measuring a felled tree in Numba (A), herbivore sampling from felled trees in Mikulcice and Toms Brook (B, C), sampling from canopy crane in Tomakomai (D, E), a tree climber accessing a tree inaccessible from the crane in Tomakomai (F), sampling of an understory tree by ladder in San Lorenzo (G), sampling from cherry picker in Lanzhot (H, I), sample sorting and caterpillar rearing in Tomakomai (J). The individuals whose faces are fully or partially visible in this figure have given written informed consent (as outlined in PLOS consent form) to publish these photos.

Plot-based approaches applied to forest vegetation have greatly benefitted plant ecology research [32]. We anticipate the study of arthropod interaction networks would benefit in equal measure [33]. We accessed canopies using tree felling, canopy crane, and cherry picker techniques (Fig 1) across biogeographic regions (Palearctic, Nearctic, Neotropical, and Australian) and forest types (tropical vs. temperate, lowland vs. montane, primary vs. secondary). We compare our plot-based methods with non-plot-based sampling and highlight the strengths and limitations of the methods for sampling mobile flightless exophytic herbivores (leaf-chewing insect larvae), endophytic herbivores (miners and gallers), and flightless invertebrate predators (spiders and ants). Our aim is to stimulate plot-based research by providing practical and reproducible sampling guidelines for the analysis of arthropod interaction networks in forest canopies. We expect i) plot-based data and non-plot-based data to provide largely different estimates of interaction network structure as non-plot-based sampling skews frequencies between rare and abundant species, ii) felling to be the most efficient method in terms of sampling effort as it allows employing large teams of field workers who can simultaneously access large parts of the canopy, iii) all three methods to provide similar acessibility to the canopy with access to over 75% of foliage.

## Materials and methods

During our operations, we took advantage of ongoing logging operations (Mikulcice, Toms Brook) and shifting agriculture (PNG sites); no plot was cleared solely for sampling. All projects were conducted in close collaboration with the local community and land owners. We obtained all research and export permits where required. Arthropods and plants from Papua New Guinea were sampled and exported under the permits nr. 070382, 070384, 080275, 010075, 011209, 011324, 012134, 014282, 0133004, 133005, and 018060 issued by Department of Environment and Conservation, Papua New Guinea, and 0139/2008, 0162/2010, and 0203/2013 issued by Forest Research Institute and Department of Forests, Papua New Guinea. Arthropods and plants from Panama were obtained and exported under the permits nr. SE/A-49-16, SE/AP-28-16, SC/AP-2-16, SEX/P-30-17, SEX/A-67-17, SEX/A-76-17 issued by Ministerio de Ambiente, Panama. The individuals whose faces are fully or partially visible in Fig 1 have given written informed consent (as outlined in PLOS consent form) to publish these photos.

**Fig 2:**
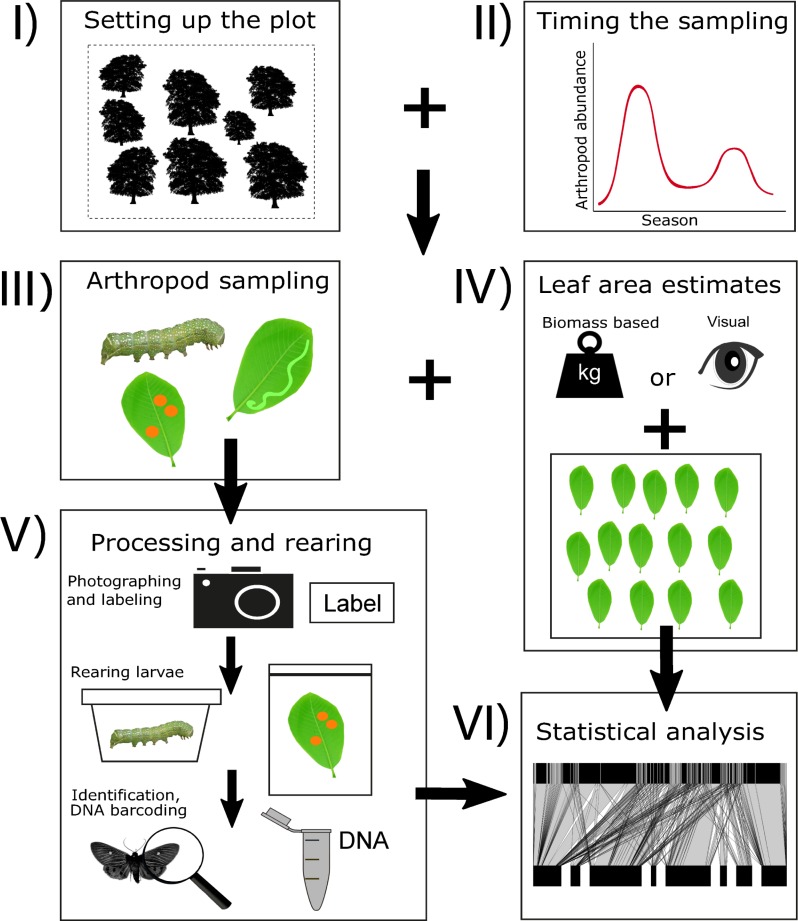
A workflow diagram for the proposed methods. The process starts with setting up the plot (I) and planning the sampling according to seasonality at a given site (II). The field work includes arthropod sampling (III) and estimation of leaf area (IV, including visual or biomass based estimates and processing of leaf frames). Sampled arthropods are then processed (V), which includes the labelling and photographing of morphospecies, rearing, and the sending of material for taxonomic identification or DNA barcoding. Finally, the data are analysed (VI).

Following a standardized protocol (Appendix 1) and workflow (Fig 2), we sampled i) lowland temperate forests in the Czech Republic (Mikulcice, Lanzhot), Japan (Tomakomai), and USA (Toms Brook); ii) lowland tropical forests in Panama (San Lorenzo) and Papua New Guinea (hereafter PNG; Wanang); and iii) highland tropical forests in PNG (Numba, Yawan) (Table 2, S1 Table).

**Table 2:** Sampling site characteristics. Forest type (Trop—tropical., Temp—temperate), lowland (90–230 m a.s.l.), highland (700–1800 m a.s.l.), primary (P), and secondary (S) forests); Maximum tree height (m); Plots (number and size of sampled plots); Method of sampling; mean Number of stems with DBH≥5 cm per 0.1 ha (±SD); mean Sampled leaf area (m^2^) per 0.1 ha (±SD); mean number of Leaf-chewing larvae per 0.1 ha (±SD); mean number of Active mines per 0.1 ha (±SD); mean Area-based sampling effort per 0.1 ha (ASE, person-hours; ±SD); mean Resource-based sampling effort (RSE, person-hours per 1 m^2^ of foliage; ±SD); mean Accessibility (% of foliage accessed; ±SD); average Team size in the field and lab combined; and Sampling period (month and year). See S2 Table for data by individual plots and all arthropod groups.

Site	Forest type	Maximum tree height (m)	Plots	Method	Number of stems	Sampled leaf area (m^2^)	Leaf-chewing larvae	Active leaf mines	ASE (person-hours)	RSE (person-hours)	Accessibility (%)	Team size	Sampling period
Tomakomai (JPN)	Temp. lowland (P)	22.8	2 x 0.1ha P	Crane	92 ±16	1,219 ±116	8,300 ±825	385 ±196	1,330 ±178	1.10 ±0.25	82.0 ±0.1	7	May-Aug 14; May-Aug 15
Lanzhot (CZE)	Temp. lowland (P)	45.0	2 x 0.1ha P	Cherry picker	29 ±6	1,208 ±194	4,891 ±576	148 ±60	1,128 ±305	0.92 ±0.10	89.3 ±6.3	8	May-Aug 13; May-Aug 14; May 15
Mikulcice (CZE)	Temp. lowland (P)	33.6	1 x 0.1ha P	Felling	53	1,137	2,352	2717	1,512	1.33	83.4	10	May—June 13
Toms Brook (USA)	Temp. lowland (P)	30.7	2 x 0.1ha* P	Felling	81 ±18	1,793 ±132	2,608 ±428	564 ±470	1,604 ±326	0.89 ±0.12	76.5 ±1.0	7	Apr-Aug 16; Apr-Aug 17
San Lorenzo (PAN)	Trop. lowland (P)	35.0	3 x 0.1ha P	Crane	91 ±6	2,023 ±303	808 ±754	1,007 ±965	2,404 ±416	1.19 ±0.03	83.3 ±5.5	5	May 16-Apr 17
Wanang (PNG)	Trop. lowland (P+S)	74.2	1 x 1.0 ha (P) 1 x 1.0 ha (S)**	Felling	120 ±30	3,377 ±1050	1,354 ±705	185 ±85	1,880 ±474	0.58 ±0.14	82.9 ±4.0	21	Jan 06-Nov 07
Numba (PNG)	Trop. highland (P+S)	49.6	2 x 0.2 ha (P) 1 x 0.2 ha (S)**	Felling	143 ±17	3,658 ±1403	1,118 ±321	60 ±32	1,800 ±642	0.52 ±0.19	81.6 ±3.5	16	May 13-Jun 14
Yawan (PNG)	Trop. highland (P+S)	65.7	4 x 0.2 ha (P) 5 x 0.2 ha (S)**	Felling	133 ±62	3,591 ±620	1,103 ±862	199 ±152	1,183 ±488	0.33 ±0.14	82.9 ±4.0	16	Jul 10-Dec 12

* one of the 0.1 ha plots consisted of a 0.06 ha plot and a 0.04 ha plot separated by a 50 m gap

** these plots were divided into 0.1 ha plots for the purpose of the analysis

### Setting up the plot

At each location, we selected 0.1 ha plots with a vegetation structure and species composition typical for local broadleaf forests (Table 2, S1 and S2 Tables). In Wanang, Numba, and Yawan, plots were larger and subdivided into 0.1 ha sections (Table 2). Forest edges, plantations, stands with non-native vegetation, and large gaps were all avoided, as were steep slopes and swampy areas (for technical and safety reasons). We took GPS coordinates of all plot corners and used measuring tape or laser range finders to set up the plot and map all plants with DBH ≥5 cm. Each stem was tagged and identified to species level. It took 2–12 hours for three people to set up a 0.1 ha plot and map 24–251 trees within. In Tomakomai (4 trees), Toms Brook (8), and San Lorenzo (17) some trees proved to be hazardous to sample or were damaged by factors beyond our control, such as a hurricane, during the sampling. These trees were replaced by conspecifics or other broadleaf trees with a similar DBH adjacent to the plot. One non-native and one coniferous tree in Toms Brook were treated in the same way.

### Timing the sampling

Arthropod abundances and species composition can vary dramatically throughout the year in seasonal forests. For example, temperate leaf-chewing insects exhibit one major peak during spring leaf-flush, and a smaller peak in late summer [12]. Furthermore, peaks in abundance may differ among arthropod guilds, for instance leaf miners, where the major peak appears to occur later than for leaf-chewers (S1 Fig, S3 Table). A single, short sampling campaign can fail to capture all arthropod groups. Therefore, we generally sampled temperate plots at a slower pace throughout the season to mitigate this undesirable effect, returning periodically to the sites in order to sample trees (i.e. typically one to several tree individuals were sampled per day depending on their canopy sizes). Sampling effort was increased during abundance peaks if they materialised. During this period, sampling was conducted whenever the weather permitted. In this way, the variation in sampling effort mirrors the variability in insect abundance, and the probability that an insect will be sampled remains constant throughout the season. We spread the sampling seasonally within each sampled tree species to avoid a bias due to an unbalanced seasonal sampling (Appendix 1). In wet tropical forests, sampling was carried out with constant effort throughout the seasons as the effects of seasonality are much less pronounced and individual species appear throughout the year [34]. However, a variable sampling strategy would be advisable in dry tropical and subtropical forests, where seasonality asserts greater influence [35]. Such intense and relatively long-term sampling required careful logistical planning. This involved negotiating the research plan well in advance with land owners, crane drivers, chainsaw operators, and local managers so as to avoid clashing with other projects at the given sites. For example, for the sampling from felled trees, we specifically sought plots of forest that were scheduled for logging and paid the loggers to cut the trees on our schedule.

### Arthropod sampling

The requirements for accessing the forest canopy and obtaining live arthropods dramatically limit the range of suitable methods for the study of quantitative arthropod interaction networks (Table 1). We sampled arthropods from felled trees, and from standing trees using canopy cranes or cherry pickers (Fig 1). Arthropods were, as far as possible, completely sampled from all trees with DBH ≥5 cm. The percentage of the canopy accessed was visually estimated for each tree (Appendix 1). We sampled on days without strong rain or wind to mitigate safety risks and lowered arthropod activity due to harsh weather. The focal arthropod groups included all live leaf-chewing insect larvae (free feeding and semi-concealed), leaf mines, galls (insects and mites), spiders, and ants (foraging and nesting; S1 Table). Some species of galls were extremely abundant, making their complete sampling impractical. In such cases, we selected 3–5 branches each with 100–500 leaves, calculated the mean number of galls per leaf per branch, and used the resulting values to estimate the total abundance on the respective tree (Appendix 1).

#### Felling

Felling trees as a standardized destructive method is only suitable when it does not contribute to net deforestation. During our operations, we took advantage of ongoing logging operations (Mikulcice, Toms Brook) and shifting agriculture (PNG sites); no plot was cleared solely for sampling. All projects were conducted in close collaboration with the local community and land owners.

Sampling began with the clearing of the understory, followed by the felling of trees with DBH ≥5cm. One tree was felled at a time, starting with the shortest. Lianas on trees were cut prior to felling in order to free up the focal tree from its neighbours. Felled individuals were directed into gaps created by previous felling. Once felled, the entire tree (trunk included) was searched and all focal arthropods hand collected, a process taking anywhere from minutes to several hours, depending on the crown size. Prompt work minimized the loss of arthropods through dispersal or predation. It also prevented the contamination by foraging ants and spiders from the ground. Using division of labour, each team member focused primarily on one arthropod group, but would also contribute to the collection of secondary groups. Trees were always fully sampled on the day of felling, and necessitated teams of 7–21 members, dependent on study site and season (Table 2).

Unlike sampling from cranes and cherry pickers, felling allows the sampling of arthropods dwelling in large branches and trunks, such as nesting ants (Table 1). At felling sites, we intensively searched every tree for ant nests and foraging ants with a team of two to three collectors, as described in Klimes et al. [16]. Foraging ants were collected first, before searching for ant nests by cutting branches, inspecting live and dead twigs, by dissecting parts of the trunk and bark, and by inspection of epiphytic aerial soil (Appendix 1).

Conversely, felling is not suitable for mobile, flying herbivores [36]. Even non-flying herbivores may become dislodged when the crown forcefully impacts the ground. If this were a serious concern, the ratio between endophytic herbivores and leaf-chewing larvae would depend on the method. However, the ratio of leaf-chewing larvae to active miners sampled in individual 0.1 ha plots did not differ among the methods (χ^2^ (2) = 2.57, p = 0.2764) when compared by linear mixed-effect models using the ‘lmer4’ R package [46], with site as a random effect.

#### Crane

We sampled arthropods from canopy cranes in Tomakomai and San Lorenzo. In Tomakomai, the crane is 25 m high, covers ca 0.5 ha of forest, and is operated by researchers from the gondola. In San Lorenzo, the crane is operated by a driver. The maximum accessible height from the gondola is 40.5 m. The crane covers almost 1.0 ha of tropical forest [37].

There were 4–7 team members working in the field, typically including 2 members sampling from the crane (canopy team), 1–2 members sorting samples on the ground (ground team), and possibly 1–2 members accessing larger mid-story trees by climbing (climbing team). The canopy team sampled branches starting at the tip and working towards the base, in order to minimize arthropod loss during sampling. Arthropods were sampled by beating onto a beating tray, followed by a visual search and hand collection of any remaining arthropods. The canopy team was assisted by an additional member during periods of peak arthropod abundance. The samples were regularly delivered to the ground team for sorting.

Sampling from the crane was augmented with other methods. The canopy team accessed understory trees from ladders. Step ladders were ideal for sampling 3–5 m tall trees. For sampling at heights up to 8 m, or on sloped terrain, modular ladder poles were more efficient and stable. In addition, more complex forest architecture, as in San Lorenzo, required the climbing team. Using a single rope technique, they accessed those mid-story trees inaccessible from the gondola or ladders (Fig 1).

#### Cherry picker

A cherry picker (an elevated truck-mounted work platform) was employed in Lanzhot. The 20 ton vehicle was transported by truck to the site, thus necessitating a forest access road. We used a Platform GENIE Z-135/70 JRT (Genie Industries, Redmond, WA, USA), which was equipped with a retractable arm enabling canopy access up to 43 m. The arm was operated by researchers directly from the basket. This four-wheel drive model can operate on gravel or clay forest roads, but not on off-road terrain. Plots were set up along a forest road with a firm dirt surface (~4 m wide, and completely covered by forest canopy) in order to provide good access to the plot from a single straight trajectory and to avoid having to manoeuvre the cherry picker between trees. Two team members sampled trees starting from the base and working towards the treetop. Arthropods were sampled using a beating tray combined with hand collection of any remaining individuals, before a final manual search by both workers. Samples were delivered to the ground team for processing before transportation to the laboratory. There were 2–6 people processing samples in the ground team, depending on insect abundance.

### Leaf area estimates

We calculated the leaf area of sampled trees in order to standardize arthropod abundance and allow cross-site comparisons (Appendix 1).

At the felled sites, we quantified leaf biomass directly by defoliating each tree and weighing the fresh foliage. Mature and young leaves were sampled and weighed separately immediately following herbivore sampling. Care was taken that only leaves, with no other plant parts such as twigs and flowers, were sampled. At Mikulcice and Toms Brook sites, where team size was limited, only 50% or 25% of the canopy was defoliated on the largest trees and the results extrapolated to 100%. This measure was taken to ensure the complete sampling of large trees on the day of felling.

At the crane and cherry picker sites, defoliating trees and weighting the biomass was not possible. Instead, we visually estimated the number of young and mature leaves on standing trees. These estimates were conducted separately for every branch sampled for arthropods. The estimates were carried out for branches with ca 500 leaves each by two persons from the canopy team. The mean value of the two estimates was taken. The branch level estimates for the given tree were then summed to give an estimate for the entire tree. This method yielded more reliable results than if estimating leaves on larger branches or whole trees.

At all sites, a random sample of leaves from each tree was then arranged on a 50 x 50 cm board with white background (the “leaf frame”) and photographed. One frame each of young and mature leaves was processed for small trees (DBH <15 cm), while at least two frames were processed for larger trees. The leaf area of each sample was then calculated using ImageJ 1.48 [38]. For felled trees, we included the weight of the sample to obtain the area to weight ratio. For the trees sampled from cranes and cherry pickers, we divided the leaf area of the sample by the number of leaves in the frame to obtain the mean area per leaf.

Finally, we calculated the total sampled leaf area for each tree using (i) the total leaf biomass and the area to weight ratio from the photographed sample for the felled trees, or (ii) the estimated total number of leaves on the tree multiplied by the mean leaf size of the photographed sample for the crane and cherry picker trees.

### Sample processing

In Tomakomai, Mikulcice, and Lanzhot, pre-sorting, photographing, and labelling of samples was done in the field by a team consisting of 1–6 members, depending on arthropod abundance (Appendix 1). This made subsequent sorting in the lab much faster. Smaller trees in Toms Brook were treated the same way. Otherwise, samples were processed entirely in the laboratory.

We assigned all leaf-chewing insect larvae, galls, and mines to morphospecies according to their morphology [21]. Each morphospecies was given a unique code name and was photographed. We preferred to assign initial morphotypes *de novo* per each individual tree sampled instead of using a complex system of morphospecies across all trees within the plot or even across multiple plots (Appendix 1). This approach is rapid and resistant to errors as even incorrect morphotyping does not generate false host plant records. It requires a second step where individual morphospecies are cross-referenced across all trees on completion of sampling. It is suitable for taxonomically poorly known and species diverse samples, where per-guild richness for an entire plot could reach hundreds of morphospecies.

We reared larval insect herbivores to adults or parasitoids (Appendix 1). Only in Toms Brook, where insect taxonomy and host associations are well known, were leaf-chewing larvae immediately stored in ethanol due to the overwhelming logistics of rearing all. We preserved larvae that died during rearing, the larvae from Toms Brook, spiders, and representative samples of all ant castes from each nest or foraging event in vials with 95% ethanol for subsequent DNA barcoding. The results of DNA barcoding along with reared adults are being used to refine morphospecies concepts and assign final identifications [21, 23, 39–41]. See Data Accessibility section for details on the publicly available sequences.

### Statistical analysis: Comparing methodological approaches

Non-plot-based studies typically focus on i) abundant tree species or ii) a taxonomically/phylogenetically representative selection of species e.g. [42, 43, 44]. In order to compare plot-based and non-plot-based methods, we derived both types of data from plot-based data on plant-caterpillar interactions in 0.8 ha of PNG highland primary rainforest Yawan [23]. This dataset was chosen because it is species-rich, the caterpillar species exhibit various levels of host specificity, and were already identified to an acceptable level. Only living trees identified to species and caterpillar species/morphospecies with confirmed host associations were included. The pruned dataset, representing 0.8 ha of primary forest (eight x 0.1 ha plots), included 113 tree species and 186 caterpillar morphospecies.

We computed network statistics and structure from the Yawan primary plots, and compared them with networks comprised of i) the most abundant (in terms of amount of foliage based on leaf area calculations) tree species and ii) a taxonomically representative selection of primary tree species including all tree families that had at least 200 m^2^ of foliage sampled for arthropods.

For type (i) networks, we combined all 0.1 ha plots to represent a larger patch (0.8 ha) of rainforest and ranked tree species in order of decreasing amount of total foliage. We then selected the species whose cumulative total foliage represented 20%, 40%, 60%, and 80% of the total foliage of the 0.8 ha patch (3, 7, 15, and 31 species, respectively). In each threshold category, we rarefied the foliage amount of each species (F_sp_) to equal the average total foliage of a 0.1 ha plot divided by the respective number of tree species (F_thresh_). This was achieved by randomly selecting individual trees until F_sp_ > = F_thresh_. The final trees (T_f_) are only partially sampled of their caterpillars (C_f_) so that F_sp_ = F_thresh_ (if we use 0.25T_f_ then we take 0.25C_f_ randomly selected caterpillars, rounded to the nearest integer). The individual trees and the caterpillars found on them made up the networks from which statistics were computed. The process was repeated 100 times for each category to account for the random tree (and caterpillar) selection.

For type (ii) networks (taxonomical selection), we limited the dataset to tree species and families that had at least 200 m^2^ of foliage sampled (200 m^2^ equals ca 0.7% of the total foliage in the 0.8 ha primary forest patch). We selected the most abundant species per family in terms of leaf area (19 species and families selected). With this tree selection, rarefaction then proceeded as per type (i) networks.

We focused our comparison between plot-based and non-plot-based networks on: i) connectance (realised proportion of possible links), ii) web asymmetry (balance between numbers of species in the two levels; positive values indicate higher proportion of higher trophic level species), iii) nestedness (temperature of the matrix; 0 means high nestedness, 100 means chaos), iv) species richness of caterpillars, v) weighted generality (mean effective number of host species per caterpillar species), and vi) weighted vulnerability (mean effective number of caterpillar species per host species) as defined and computed in ‘bipartite’ package [45]. The network parameters were compared using 95% confidence intervals.

Furthermore, we compared the efficiency of each sampling method we used across the plots. We expressed the method efficiency as i) **Foliage accessibility** per plot (the average percentage of accessible foliage), ii) **Area-based sampling effort** (**ASE**) required to sample each 0.1 ha plot (in person-hours), and iii) **Resource-based sampling effort** (**RSE**) required to sample 1 m^2^ of foliage (in person-hours). Only time spent on sample collection and sorting in the field was counted towards the sampling effort. Workers helping with logistics (chainsaw operators or the crane driver in Panama) were excluded. We modelled the relationship between these components of sampling efficiency and the sampling **Method** (felling, crane, cherry picker), **Forest type** (temperate, tropical lowland primary, tropical lowland secondary, tropical highland primary, tropical highland secondary), the **Number of stems** (DBH ≥ 5 cm) per plot, and **Sampled leaf area** using linear mixed-effect models as implemented in the R package ‘lmer4’ [46]. **Foliage accessibility** was arcsine-transformed and **sampling effort** log-transformed. We used **Site** as a random factor in all mixed-effect models (S2 Table). Model simplification by forward selection was employed to produce the most parsimonious model based on Akaike’s Information Criterion (AIC). All analyses were performed in R software version 3.4.0 [47].

## Results

In total, we sampled focal arthropod groups from 5.3 ha of forest, representing 6,280 trees and 167,744 m^2^ of foliage (Table 2). We sampled 89,243 leaf-chewing larvae, 14,547 active mines, 135,446 abandoned mines, 28,698 spiders, 35,343 ant individuals, 3,487 ant nests, and sampled or estimated abundance of 2,963,942 insect and mite galls (S2 Table).

**Fig 3:**
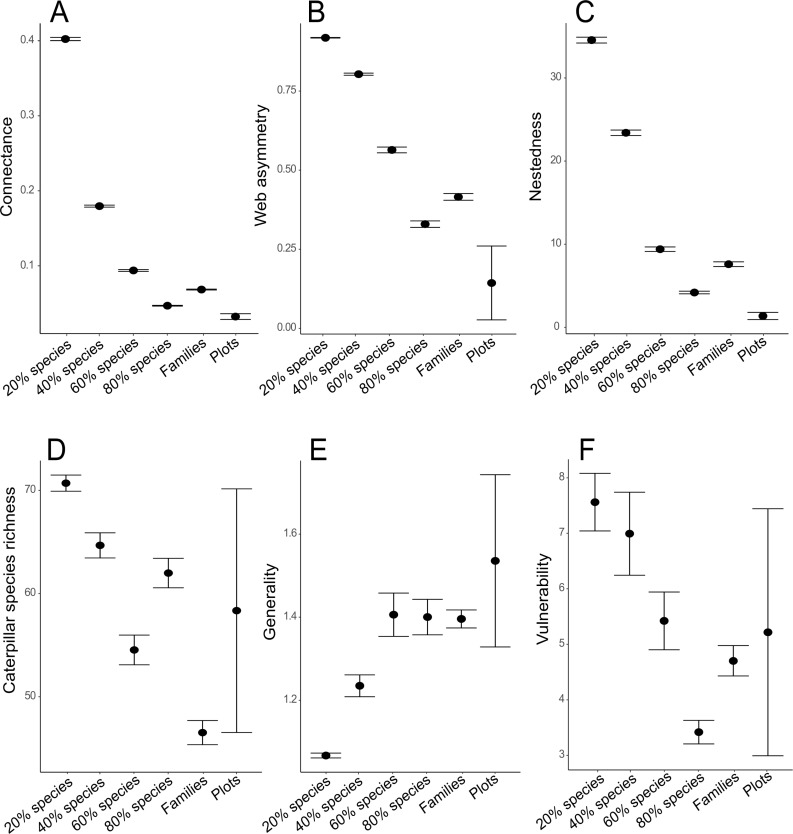
Parameters of plant-caterpillar interaction networks based on the plot-based data (Plots) and simulated non-plot-based data where individual tree species were sampled with equal effort. The simulated data represent a non-plot-based approach focusing on locally abundant tree species representing a certain amount of the foliage in the forest (20, 40, 60, or 80% species) or a representative selection of tree families (Families). The results are based on Yawan primary forest dataset from Redmond et al. [23]. The compared network parameters include connectance (A), web asymmetry (B), nestedness (C), species richness of caterpillars (D), weighted generality (E), and weighted vulnerability (F). All simulated datasets were rarefied to the average leaf area of a 0.1 ha plot. All rarefactions were repeated 100-times. Points show mean. Bars show 95% confidence intervals.

All non-plot-based data types showed highly biased network structure towards higher connectance, higher web asymmetry, and higher nestedness temperature when compared to the plot-based data (Fig 3). Non-plot-based data using the most abundant tree species representing 20% of the foliage in the local forest had the highest caterpillar richness while those using a taxonomically representative selection of tree families had the lowest caterpillar richness. Non-plot-based data using the most abundant tree species representing 20% and 40% of the foliage in the local forest showed lower generality than the plot-based data. Differences in vulnerability were less pronounced mainly because of the high variability in plot-based data. However, vulnerability was highest in the data using the most abundant tree species representing 20% and 40% of the foliage in the local forest.

On average, **Foliage accessibility** was 82.5% ±3.9% (mean ±SD) foliage in felled plots, 82.7% ±3.3% foliage in plots sampled by canopy crane, and 89.3% ±6.3 foliage in plots sampled by cherry picker (S2 Fig). **Foliage accessibility** correlated with **Method** (χ^2^ (2) = 6.91, p = 0.0254). The optimum model, after simplification, included the fixed effects **Forest type** (highest in lowland and highland secondary tropical forests), **Method** (highest from the cherry picker), **Number of stems** (positive correlation), and **Sampled leaf area** (negative correlation) (χ^2^ (8) = 64.02, p < 0.0001) (S4 Table).

The average **ASE** required to sample a 0.1ha plot was 1583 ±579 person-hours (mean± SD) for felled trees, 1867 ±673 for sampling by canopy crane, and 1128 ±305 for sampling by cherry picker. **Method** did not have a significant effect on **ASE** (χ^2^ (2) = 1.49, p = 0.4740). The optimum model that explained differences in **ASE** included the fixed effects **Number of stems** (positive correlation) and **Forest type** (highest in lowland primary tropical forests) (χ^2^ (5) = 95.24, p < 0.0001; S4 Table).

The average **RSE** to sample 1 m^2^ of foliage was 0.51 ± 0.24 (mean± SD) person-hours for sampling felled trees, 1.14 ±0.15 for sampling by canopy crane, and 0.92 ±0.10 for sampling by cherry picker. **Method** did not have a significant effect on **RSE** (χ^2^ (2) = 3.52, p = 0.1722). The optimum model explaining differences in **RSE** included the fixed effects **Number of stems** (positive correlation), **Sampled leaf area** (negative correlation), and **Forest type** (highest in temperate forests) (χ^2^ (6) = 80.75, p < 0.0001; S4 Table).

## Discussion

We propose a plot-based approach to studying arthropod interaction networks, using three methods for sampling a continuous area of forest canopy. Plot-based standardisation means that frequent associations can be distinguished from those that are casual or rare [48, 49]. Focusing on a selection of abundant tree species or representative families sampled with a standardized sampling effort skews the proportions between rare and common interactions. As expected, this resulted in higher connectance, high web asymmetry and higher nestedness in the simulated non-plot-based data. This is not surprising as all these parameters are linked to the network size, which has been reduced under the selective sampling scenario. In addition to reducing network size, non-plot-based sampling focused on abundant or phylogenetically distinct hosts can affect the patterns recovered in host specificity and diversity. This is because such hosts typically harbor distinct arthropod communities. Locally abundant tree species tend to harbor higher diversity of herbivores than rarer hosts [50]. Focusing on such hosts can lead to over-estimations of diversity. On the other hand, hosts from isolated or chemically distinct families can have relatively species poor herbivore communities [51]. Emphasizing such hosts in the data can lead to under-estimations of diversity. Also, many herbivores are shared between congeneric or confamilial hosts while the amount of shared herbivores decreases with the host phylogenetic distance [44]. Specificity of interactions can thus be biased in datasets that include skewed proportions of such hosts although this trend was not particularly pronounced in our simulated data.

**Fig 4:**
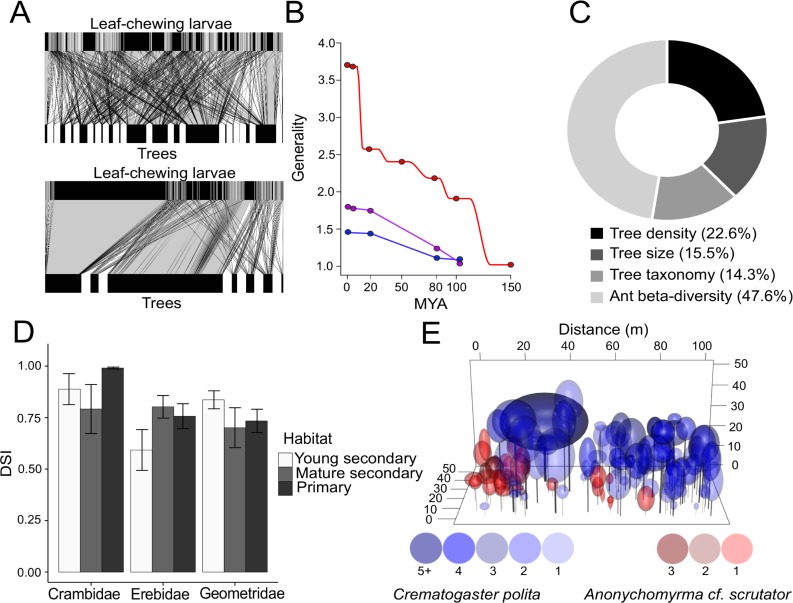
Example results from plot-based sampling. Construction of comparable quantitative interaction networks (A: plant-caterpillar food-webs from two 0.1 ha plots with contrasting herbivore and tree diversity; based on data from Volf et al. [21]). Such networks can be used to quantify effects of plant traits or phylogeny on arthropod communities (B: effects of host phylogeny on caterpillar food-webs quantified by change in generality from herbivore data collated according to the time of divergence of their hosts (in Tomakomai (red), Lanzhot (purple), Mikulcice (blue)); based on data from Volf et al. [21]). The relative contribution of such effects can be decomposed, allowing the prediction of arthropod community composition (C: the proportional difference in total ant species richness between primary and secondary forest in Wanang due to the effects of vegetation composition and species turnover; based on data from Klimes et al. [28]). Furthermore, standardized measures of herbivore specialization can be made, enabling meaningful comparisons across habitats and taxa with variable phylogenetic diversity and plant abundance (D: mean Distance Based Specialisation Index (DSI*) +/- SE for Crambidae, Erebidae, and Geometridae along a successional gradient in Yawan; based on data from Redmond et al. [23]. Finally, we can analyse spatial patterns in canopy arthropod communities (E: distribution of tree canopy nest density in the two most abundant ant species in 0.4 ha of Wanang forest (only trees with nests are shown); based on Klimeš and Mottl (*unpublished data*)).

Plot-based sampling provides a robust description of the community structure as one can assume that the interactions are completely censused for the proportion of the canopy successfully sampled (Fig 4). One can then test and improve the performance of models that predict trophic interactions in real communities by decomposing the effects of abundance, plant characteristics and arthropod community composition [28, 41]. Derived food-web metrics are comparable on a common area basis, and may identify processes shaping communities of canopy arthropods across various habitats, ecosystems, or geographic regions [21].

Plot-based analyses thus represent an ideal counterpart to those based on a stratified selection of focal species sampled with an equal sampling effort. Methods that employ equal sampling effort are advantageous for studying the host specialization of herbivores, the effects of host traits on herbivore communities, or insect-plant interactions in a phylogenetic framework e.g. [14, 30]. However, modern methods enable the measurement of host specialization with respect to host phylogeny or chemical similarity in plot-based data also [52]. Furthermore, a plot-based approach can be used to investigate spatial distribution of arthropods across the forest canopy and their impact on competitors and other trophic levels. This is important, for instance, when considering competition among ants where canopy connectivity and structure play important roles in forming ant communities [16]. Furthermore, herbivores may have density-dependent effects on plant survival that need to be studied in a spatially explicit framework [53].

One limitation of plot-based sampling methods is that the logistical challenges necessitate relatively large teams and overall effort. Despite our expectations, however, all methods demanded comparably high sampling effort, with none being significantly more efficient. Such prerequisites stem from the need to census all parts of the canopy, including those difficult to access, in order to reconstruct truly quantitative interaction networks [7, 21]. Foliage accessibility positively correlated with the number of stems in the plot, probably because many of the trees in densely vegetated plots were small and easier to access. On the other hand, the number of stems within a plot increased both types of sampling effort that we quantified. ASE (total effort per 0.1ha plot) was highest in lowland primary tropical forests characterized by relatively high stem density and large trees difficult to sample. RSE (effort per 1 m^2^ of foliage) was highest in temperate forests. This may be because arthropod density is generally higher in temperate forests [5], especially during the spring abundance peak.

High effort per site prevented a rigorous methodological comparison where the same forest is sampled by all three methods. All methods enabled access to over 80% of the foliage. But the unbalanced distribution of methods may be one reason why the cherry picker appeared to provide better access to the canopy than felling or cranes. Similarly to Corff and Marquis [20], we operated the cherry picker in almost optimal conditions in temperate forest where plots were close to an access road and the trees could be accessed from a straight trajectory. Operating in less favourable conditions would dramatically decrease foliage accessibility or require employing additional methods. Sampling from cranes also had to be supplemented by other techniques at both our crane sites. While sampling by other techniques represented a small proportion of sampling effort in the temperate Tomakomai forest, it considerably increased the sampling effort in San Lorenzo tropical rain forest. In San Lorenzo, only 49% of the trees (representing 58% of the foliage sampled) were accessed solely by crane.

Each method also has its own specifics unrelated to its overall efficiency. Felling generally requires larger teams [22, 23] as felled trees need to be sampled immediately. Cranes and cherry pickers allow proceeding at a slower pace with a smaller team e.g. [12, 20]. The three methods are also not completely comparable in terms of the sampled arthropod groups. All were suitable for sampling endophytic and exophytic non-flying arthropods. Less mobile flying herbivores, such as aphids or psyllids, were also well represented in our samples, although they were not the focus of our study. Felling was the only method which enabled sampling of nesting ants, which can represent an important proportion of the canopy arthropods [16]. Quantitative sampling of highly mobile macroscopic arthropods (adult beetles, flies or true bugs) was not possible by these methods, although they were better represented in crane and cherry picker samples.

Other methods, such as fogging, may be more suitable for surveying highly mobile arthropods [25]. Such methods can also strongly reduce the required team size and effort. To assess trophic interactions, however, they would need to be combined with a massive barcoding effort so the sampled arthropods could be reliably assigned to their host-plants. A molecular approach to assessing trophic interactions is becoming increasingly popular [15] and can be especially useful in well studied or less diverse communities. However, the approach may face identification limitations in diverse communities that include a high proportion of closely related and/or hybridizing hosts. Indeed, standard barcode markers may fail to provide a sufficient resolution for such hosts unless combined with specifically selected ones [54]. The implementation of such methods for large scale plot-based sampling should, therefore, be carefully considered. Furthermore, the sampling of endophytic or semi-concealed herbivores and ant nests would require the employment of additional methods.

We suggest that a global network using the methods described for area-based sampling would provide important insights into the processes of food web assembly and dynamics [33]. To that end, we propose a network of permanent plots where canopy arthropods and their interactions would be censused by non-destructive sampling. The network of permanent plots could benefit from collaboration with the global network of ForestGEO plots [32] which generates major insights into forest community ecology. We suggest that plots of 0.1 ha are an appropriate size to be sampled from cranes or cherry pickers, and which allow for repeat surveys, while keeping the required effort manageable. A single 0.1 ha plot census can yield information on more than 100,000 canopy arthropods and their interactions, thus the potential to make significant contributions to arthropod ecology research is huge.

The network of permanent plots should ideally be augmented by a larger network of temporal plots to be sampled by felling. Despite a slight revival in canopy crane construction [55], such platforms are still missing from vast regions, including Africa and North America. Similarly, opportunities for the use of cherry pickers remain limited in many forests. The sampling of 0.1 ha plots by felling thus seems to be the only widely applicable option in many regions. These plots could be highly replicated and ideally adjacent to the ForestGeo plots.

Sampling canopy arthropods by felling can become a salvage sampling strategy to obtain data on arthropod communities being lost due to ongoing deforestation. There has been considerable activity in the past decade focused on constructing large-scale experiments, such as planting forest stands of a given richness [56], or manipulation of landscape fragmentation [57], which deepen our understanding of how ongoing changes in forest structure affect ecological interactions. However, ecologists have been slow to take advantage of ongoing logging operations, urban development, or shifting agriculture for destructive arthropod and plant sampling to salvage the data. Yet, such data in combination with data from permanent plots would enable the exploration of trends in arthropod networks along major environmental gradients [23]. Furthermore, the detailed data obtained by our methods could be used for modelling forest composition and arthropod interactions. Combining such models with high-throughput methods, such as remote sensing, that allow forest composition to be assessed may enable us to predict the basic characteristics of plant-arthropod interactions over large spatial scales [58]. Ultimately, the application of the outlined methods could lead to high-impact results with far-reaching consequences, such as the prediction of the effects of forest degradation on forest arthropod communities, and the identification and preservation of arthropod diversity hotspots in the world’s forests.

## Supporting information

S1 Fig Seasonal trends in abundance of leaf chewing larvae and active miners. (DOCX)

S2 Fig Foliage accessibility. (DOCX)

S1 Table Site characteristics. (DOCX)

S2 Table Characteristics of individual 0.1 ha plots and number of arthropods sampled. (DOCX)

S3 Table Monthly trends in abundance of caterpillars and active miners. (DOCX)

S4 Table Variables with a significant effects on Foliage accessibility, Area-based sampling effort, and Resource-based sampling effort as selected by forward selection in linear mixed effect models. (DOCX)

S5 Table List of staff, interns, students, volunteers, and local assistants who helped with the sampling. (DOCX)

S1 Appendix Sampling protocols. (PDF)
