# Supplementary material for: Quantitative assessment of plant-arthropod interactions in forest canopies: A plot-based approach
Source: PLoS One. 2019 Oct 23;14(10):e0222119. doi: 10.1371/journal.pone.0222119 (PMC6808442; doi:10.1371/journal.pone.0222119)
Supplement: S1 Fig — (DOCX) [file pone.0222119.s001.docx]

**Quantitative assessment of arthropod-plant interactions in forest canopies: a plot-based approach**

Martin Volf, Petr Klimeš, Greg Lamarre, Conor Redmond, Carlo L. Seifert, Tomokazu Abe, John Auga, Kristina Anderson-Teixeira, Yves Basset, Saul Beckett, Philip T. Butterill, Pavel Drozd, Erika Gonzalez-Akre, Ondřej Kaman, Naoto Kamata, Benita Laird-Hopkins, Martin Libra, Markus Manumbor, Scott E. Miller, Kenneth Molem, Ondřej Mottl, Masashi Murakami, Tatsuro Nakaji, Nichola S. Plowman, Petr Pyszko, Martin Šigut, Jan Šipoš, Robert Tropek, George Weiblen, and Vojtech Novotny


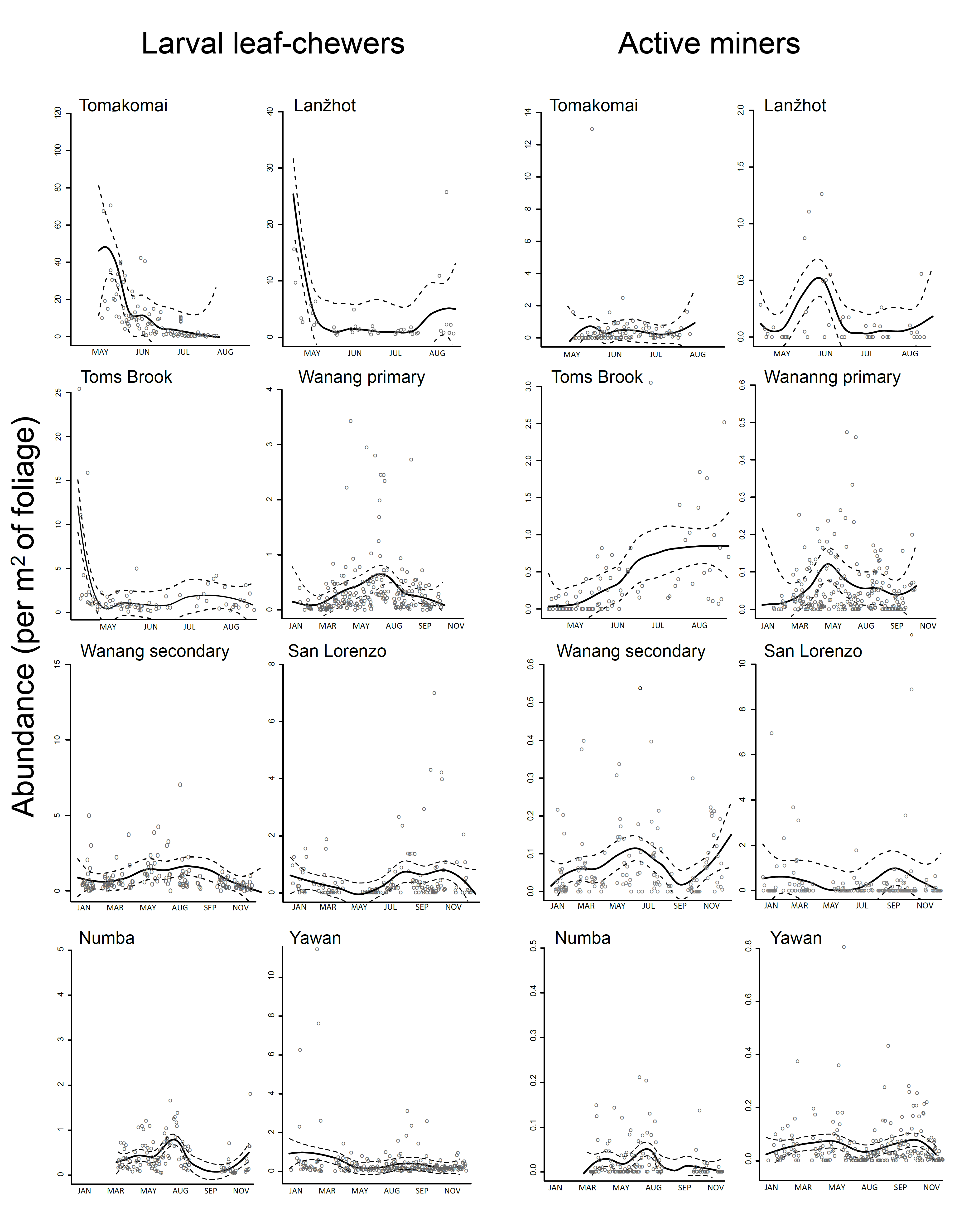


**S1 Fig.** Seasonal trends in abundance of leaf chewing larvae and active miners across the plots sampled for multiple months (Tomakomai, Lanzhot, Toms Brook, San Lorenzo, Wanang, Numba, Yawan). The data points represent number of caterpillars and active miners per 1 m^2^ of foliage on individual days of sampling. The seasonal trend was modelled with a loess smoother (solid line). Dashed lines show confidence intervals. The abundance was standardized by leaf area. Data from individual 0.1 ha plots sampled at the listed sites were combined. The data from Wanang primary and secondary forest plots were kept separate to illustrate possible differences between primary and secondary forest. Two outlier data points with leaf-chewer abundance of 226 and 18 are not shown in the case of Tomakomai and Wanang secondary, respectively. One outlier data point with active miner abundance of 25 is not shown in the case of San Lorenzo.
