# Supplementary material for: Quantitative assessment of plant-arthropod interactions in forest canopies: A plot-based approach
Source: PLoS One. 2019 Oct 23;14(10):e0222119. doi: 10.1371/journal.pone.0222119 (PMC6808442; doi:10.1371/journal.pone.0222119)
Supplement: S2 Fig — (DOCX) [file pone.0222119.s002.docx]

**Quantitative assessment of arthropod-plant interactions in forest canopies: a plot-based approach**

Martin Volf, Petr Klimeš, Greg Lamarre, Conor Redmond, Carlo L. Seifert, Tomokazu Abe, John Auga, Kristina Anderson-Teixeira, Yves Basset, Saul Beckett, Philip T. Butterill, Pavel Drozd, Erika Gonzalez-Akre, Ondřej Kaman, Naoto Kamata, Benita Laird-Hopkins, Martin Libra, Markus Manumbor, Scott E. Miller, Kenneth Molem, Ondřej Mottl, Masashi Murakami, Tatsuro Nakaji, Nichola S. Plowman, Petr Pyszko, Martin Šigut, Jan Šipoš, Robert Tropek, George Weiblen, and Vojtech Novotny


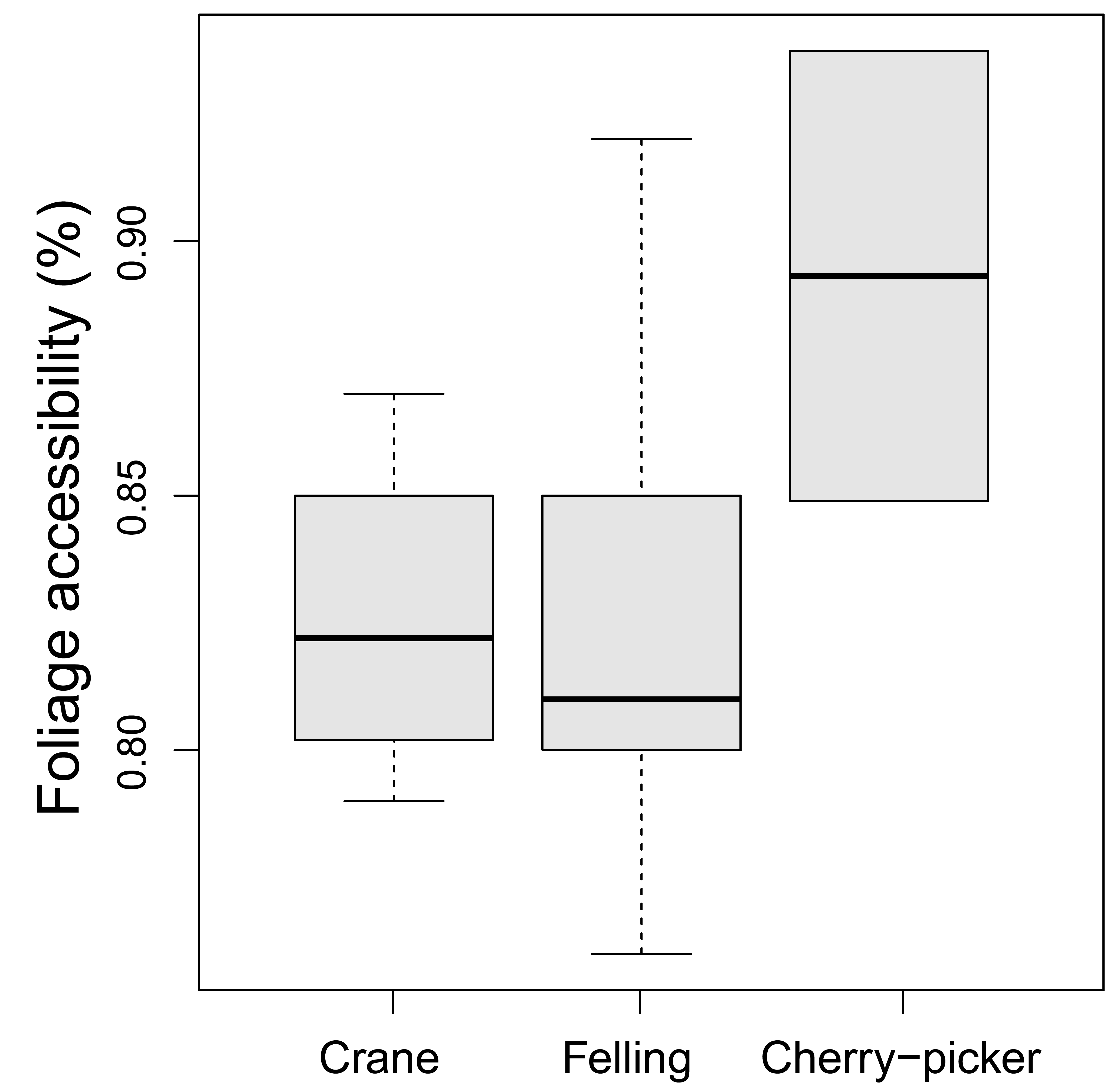


**S2 Fig.** Foliage accessibility (% of foliage possible to access in individual 0.1 ha plots) facilitated by individual methods. Canopy accessibility was correlated to the used method (χ^2^ (2) = 6.91, p = 0.0254). The highest accessibility was achieved by the cherry-picker, which operated in optimal conditions of a temperate forest.
