# Supplementary material for: Quantitative assessment of plant-arthropod interactions in forest canopies: A plot-based approach
Source: PLoS One. 2019 Oct 23;14(10):e0222119. doi: 10.1371/journal.pone.0222119 (PMC6808442; doi:10.1371/journal.pone.0222119)
Supplement: S1 Table — (DOCX) [file pone.0222119.s003.docx]

**Quantitative assessment of arthropod-plant interactions in forest canopies: a plot-based approach**

Martin Volf, Petr Klimeš, Greg Lamarre, Conor Redmond, Carlo L. Seifert, Tomokazu Abe, John Auga, Kristina Anderson-Teixeira, Yves Basset, Saul Beckett, Philip T. Butterill, Pavel Drozd, Erika Gonzalez-Akre, Ondřej Kaman, Naoto Kamata, Benita Laird-Hopkins, Martin Libra, Markus Manumbor, Scott E. Miller, Kenneth Molem, Ondřej Mottl, Masashi Murakami, Tatsuro Nakaji, Nichola S. Plowman, Petr Pyszko, Martin Šigut, Jan Šipoš, Robert Tropek, George Weiblen, and Vojtech Novotny

**S1Table.** Site characteristics including latitude, longitude, altitude, average temperature, and annual rainfall.

| Site | Latitude | Longitude | Altitude  (m asl) | T  (°C) | Rainfall  (mm) | Reference |
| --- | --- | --- | --- | --- | --- | --- |
| Tomakomai (JPN) | 42° 43' N | 141° 34'E | 90 | 5.6 | 1,450 | [1] |
| Lanzhot (CZE) | 48° 48' N | 17° 5'E | 152 | 9.0 | 525 | [2] |
| Mikulcice (CZE) | 48°41' N | 16°56'E | 164 | 9.0 | 525 | [2] |
| Toms Brook (USA) | 38°55' N | 78°25' W | 230 | 12.7 | 970 | [3] |
| San Lorenzo (PAN) | 9°16' N | 79°58' W | 130 | 26.0 | 3,140 | [3] |
| Wanang (PNG) | 5° 14' S | 145° 4' E | 150 | 25.8 | 4,000 | [4] |
| Numba (PNG) | 5°44’ S | 145°16’ E | 700 | 22.3 | 3,000 | [5] |
| Yawan (PNG) | 6° 9' S | 146° 50' E | 1,800 | 16.2 | 3,000 | [5] |

**References**

1. Hiura T: **Estimation of aboveground biomass and net biomass increment in a cool temperate forest on a landscape scale**. *Ecological Research* 2005, **20**:271-277.

2. Volf M, Pyszko P, Abe T, Libra M, Kotásková N, Šigut M, Kumar R, Kaman O, Butterill P, Šipoš J, et al.: **Phylogenetic composition of host plant communities drives plant‐herbivore food web structure**. *Journal of Animal Ecology* 2017, **86**:556-565.

3. Anderson‐Teixeira KJ, Davies SJ, Bennett AC, Gonzalez‐Akre EB, Muller‐Landau HC, Joseph Wright S, Abu Salim K, Almeyda Zambrano AM, Alonso A, Baltzer JL, et al.: **CTFS‐ForestGEO: a worldwide network monitoring forests in an era of global change**. *Global Change Biology* 2015, **21**:528-549.

4. Vincent JB, Henning B, Saulei S, Sosanika G, Weiblen GD: **Forest carbon in lowland Papua New Guinea: Local variation and the importance of small trees**. *Austral Ecology* 2015, **40**:151-159.

5. McAlpine JR: *Climate of Papua New Guinea*: Canberra: Commonwealth Scientific and Industrial Research Organization in association with Australian National University Press; 1983.
