# Supplementary material for: Quantitative assessment of plant-arthropod interactions in forest canopies: A plot-based approach
Source: PLoS One. 2019 Oct 23;14(10):e0222119. doi: 10.1371/journal.pone.0222119 (PMC6808442; doi:10.1371/journal.pone.0222119)
Supplement: S2 Table — (DOCX) [file pone.0222119.s004.docx]

**Quantitative assessment of arthropod-plant interactions in forest canopies: a plot-based approach**

Martin Volf, Petr Klimeš, Greg Lamarre, Conor Redmond, Carlo L. Seifert, Tomokazu Abe, John Auga, Kristina Anderson-Teixeira, Yves Basset, Saul Beckett, Philip T. Butterill, Pavel Drozd, Erika Gonzalez-Akre, Ondřej Kaman, Naoto Kamata, Benita Laird-Hopkins, Martin Libra, Markus Manumbor, Scott E. Miller, Kenneth Molem, Ondřej Mottl, Masashi Murakami, Tatsuro Nakaji, Nichola S. Plowman, Petr Pyszko, Martin Šigut, Jan Šipoš, Robert Tropek, George Weiblen, and Vojtech Novotny

**S2 Table**. Characteristics of individual 0.1 ha plots and number of arthropods sampled. All living trees and climbers of DBH ≥ 5 cm with foliage and known taxonomic identity and their arthropods are included. Larval leaf-chewers refer to all free living or semi-concealed leaf-chewing insect larvae. All sampled leaf-chewing larvae, including those without confirmed host record or identification, are listed. All sampled leaf mines, including those without identification, are listed. All fungal galls and mines and galls with uncertain status (e.g. possible fungal galls and pathogen damage) were removed from the list. Gall numbers marked with an asterisk refer to insect galls only (other records include mite galls as well). Spider numbers marked with two asterisks indicate that spiders were sampled from half of the plot (i.e. 0.05 ha) only. Arthropod groups, which were not sampled or their census counts are currently not available in the respective plot are marked as NA.

| **Site** | **Plot** | **Method** | **Forest type** | **Area-based sampling effort**  **(man hours)** | **Resource-**  **Based sampling effort (man hours)** | **Foliage accessibility (%)** | **Stems**  **(DBH≥5cm)** | **Leaf area**  **(m^2^)** | **Larval leaf-chewers** | **Active**  **mines** | **Abandoned**  **mines** | **Galls** | **Spiders** | **Foraging**  **ants** | **Ant nests** |
| --- | --- | --- | --- | --- | --- | --- | --- | --- | --- | --- | --- | --- | --- | --- | --- |
| Tomakomai | Tomakomai P1 | Crane | Temperate | 1,204 | 0.93 | 83 | 81 | 1,301.00 | 8,883 | 231 | 2.086 | 527,015 | 515 | 201 | NA |
| Tomakomai | Tomakomai P2 | Crane | Temperate | 1,456 | 1.28 | 81 | 103 | 1,136.70 | 7,716 | 520 | 1.429 | 531,586 | 534 | 130 | NA |
| Lanzhot | Lanzhot P1 | Cherry-picker | Temperate | 1,344 | 1.00 | 85 | 32 | 1,346.15 | 4,484 | 106 | 5.779 | 311,063 | 5,810 | 984 | NA |
| Lanzhot | Lanzhot P2 | Cherry-picker | Temperate | 912 | 0.85 | 94 | 24 | 1,071.34 | 5,298 | 190 | 5.590 | 321,015 | 4,164 | 541 | NA |
| Mikulcice | Mikulcice P1 | Felling | Temperate | 1,512 | 1.33 | 83 | 53 | 1,137.32 | 2,370 | 2.717 | 2.041 | 398,265 | 1,230 | 943 | 79 |
| Toms Brook | Toms Brook A | Felling | Temperate | 1,835 | 0.74 | 77 | 93 | 1,886.50 | 2,910 | 232 | 641 | 402,426 | NA | 582 | 65 |
| Toms Brook | Toms Brook B | Felling | Temperate | 1,374 | 0.76 | 76 | 68 | 1,699.70 | 2,305 | 904 | 2337 | 224,286 | NA | 538 | 77 |
| Numba | Numba_PA12 | Felling | Tropical Highland Primary | 1,950 | 0.49 | 80 | 137 | 2,617.69 | 1,391 | 102 | 1591 | 942* | 949 | NA | 432 |
| Numba | Numba_PA34 | Felling | Tropical Highland Primary | 1,650 | 0.46 | 83 | 133 | 2,172.11 | 1,145 | 86 | 2220 | 615* | 1,042 | NA | 419 |
| Numba | Numba_PB12 | Felling | Tropical Highland Primary | 2,175 | 0.33 | 79 | 169 | 4,434.37 | 1,189 | 61 | 3231 | 454* | 1,666 | NA | NA |
| Numba | Numba_PB34 | Felling | Tropical Highland Primary | 2,775 | 0.34 | 78 | 145 | 6,038.78 | 1,418 | 56 | 4853 | 452* | 1,667 | NA | NA |
| Numba | Numba_S1+S2 | Felling | Tropical Highland Secondary | 1,050 | 0.34 | 84 | 121 | 3,148.80 | 1,028 | 45 | 1351 | 46* | 1,089** | NA | 136 |
| Numba | Numba_S3+s4 | Felling | Tropical Highland Secondary | 1,200 | 0.41 | 87 | 154 | 3,533.30 | 539 | 12 | 2526 | 164* | NA | NA | 115 |
| Yawan | YPA12 | Felling | Tropical Highland Primary | 1,500 | 0.25 | 78 | 114 | 4,386.80 | 1,495 | 147 | 1186 | 394* | 517 | 927 | 111 |
| Yawan | YPA34 | Felling | Tropical Highland Primary | 1,350 | 0.21 | 80 | 106 | 3,279.60 | 481 | 68 | 1078 | 211* | 77** | 1,079 | 177 |
| Yawan | YPB12 | Felling | Tropical Highland Primary | 1,050 | 0.31 | 77 | 104 | 4,223.29 | 675 | 482 | 2632 | 182* | 495 | NA | NA |
| Yawan | YPB34 | Felling | Tropical Highland Primary | 900 | 0.44 | 82 | 124 | 4,217.79 | 626 | 359 | 3812 | 260* | 814 | NA | NA |
| Yawan | YPC12 | Felling | Tropical Highland Primary | 1,200 | 0.45 | 81 | 139 | 3,931.24 | 3,601 | 266 | 876 | 171* | 483 | 400 | 118 |
| **Site** | **Plot** | **Method** | **Forest type** | **Area-based sampling effort**  **(man hours)** | **Resource-**  **Based sampling effort (man hours)** | **Canopy**  **sampled (%)** | **Stems**  **(DBH≥5cm)** | **Leaf area**  **(m^2^)** | **Caterpillars** | **Active**  **mines** | **Abandoned**  **mines** | **Galls** | **Spiders** | **Foraging**  **ants** | **Ant nests** |
| Yawan | YPC34 | Felling | Tropical Highland Primary | 1,500 | 0.36 | 81 | 117 | 3,433.94 | 1,014 | 133 | 2650 | 202* | 602 | 485 | 72 |
| Yawan | YPD12 | Felling | Tropical Highland Primary | 1,275 | 0.56 | 81 | 104 | 2,845.71 | 419 | 235 | 4742 | 202* | 452 | NA | NA |
| Yawan | YPD34 | Felling | Tropical Highland Primary | 1,350 | 0.65 | 80 | 133 | 3,754.99 | 1,886 | 411 | 4885 | 243* | 969 | NA | NA |
| Yawan | YSF12 | Felling | Tropical Highland Secondary | 1,875 | 0.39 | 91 | 251 | 3,333.99 | 1,524 | 44 | 554 | 1766* | 870 | NA | NA |
| Yawan | YSF34 | Felling | Tropical Highland Secondary | 2,175 | 0.37 | 92 | 251 | 3,333.11 | 1,071 | 36 | 630 | 652* | 692 | 605 | 70 |
| Yawan | YSG12 | Felling | Tropical Highland Secondary | 1,500 | 0.20 | 86 | 209 | 3,820.00 | 1,060 | 30 | 353 | 352* | 418 | NA | NA |
| Yawan | YSG34 | Felling | Tropical Highland Secondary | 1,725 | 0.18 | 85 | 234 | 4,701.39 | 2,619 | 125 | 1044 | 684 | 675 | 557 | 76 |
| Yawan | YSH12 | Felling | Tropical Highland Secondary | 450 | 0.22 | 84 | 60 | 2,205.64 | 399 | 60 | 711 | 127* | 167** | NA | NA |
| Yawan | YSH34 | Felling | Tropical Highland Secondary | 675 | 0.16 | 81 | 71 | 3,805.55 | 403 | 317 | 2246 | 119* | 103** | NA | NA |
| Yawan | YSJ12 | Felling | Tropical Highland Secondary | 750 | 0.22 | 86 | 81 | 3,403.85 | 783 | 134 | 1737 | 207* | 456** | NA | NA |
| Yawan | YSJ34 | Felling | Tropical Highland Secondary | 525 | 0.23 | 81 | 83 | 3,331.01 | 693 | 183 | 1872 | 273* | 901 | NA | NA |
| Yawan | YSK12 | Felling | Tropical Highland Secondary | 600 | 0.41 | 80 | 73 | 2,741.99 | 452 | 76 | 572 | 82* | 542 | NA | NA |
| Yawan | YSK34 | Felling | Tropical Highland Secondary | 900 | 0.54 | 76 | 136 | 3,894.35 | 663 | 470 | 2270 | 297* | 799 | NA | NA |
| Wanang | WP1 | Felling | Tropical Lowland Primary | 1,837 | 0.70 | 79 | 123 | 3,486.88 | 397 | 214 | 3.264 | NA | NA | NA | NA |
| Wanang | WP2 | Felling | Tropical Lowland Primary | 2,285 | 0.63 | 81 | 124 | 3,792.40 | 1,431 | 167 | 2.333 | NA | NA | NA | NA |
| Wanang | WP3 | Felling | Tropical Lowland Primary | 2,444 | 0.48 | 80 | 135 | 4,715.49 | 1,260 | 177 | 1.923 | NA | NA | NA | NA |
| Wanang | WP4 | Felling | Tropical Lowland Primary | 2,397 | 0.60 | 80 | 139 | 4,179.61 | 1,128 | 244 | 1.578 | NA | NA | NA | NA |
| Wanang | WP5 | Felling | Tropical Lowland Primary | 2,284 | 0.49 | 82 | 118 | 4,649.52 | 1,593 | 109 | 3.497 | NA | NA | NA | NA |
| Wanang | WP6 | Felling | Tropical Lowland Primary | 2,519 | 0.51 | 81 | 135 | 4,008.64 | 918 | 146 | 2.703 | NA | NA | NA | NA |
| Wanang | WP7 | Felling | Tropical Lowland Primary | 2,282 | 0.46 | 82 | 154 | 4,397.18 | 717 | 209 | 7.892 | NA | NA | 4,164 | 279 |
| Wanang | WP8 | Felling | Tropical Lowland Primary | 2,027 | 0.49 | 77 | 109 | 4,448.03 | 550 | 219 | 2.654 | NA | NA | 4,218 | 142 |
| Wanang | WP9 | Felling | Tropical Lowland Primary | 2,014 | 0.75 | 80 | 115 | 2,386.43 | 698 | 174 | 6.960 | NA | NA | 3,491 | 251 |
| Wanang | WP10 | Felling | Tropical Lowland Primary | 2,159 | 0.90 | 85 | 129 | 2,219.53 | 1,223 | 362 | 6.462 | NA | NA | 9,911 | 197 |
| Wanang | WS1 | Felling | Tropical Lowland Secondary | 1,779 | 0.72 | 87 | 144 | 2,540.76 | 1,203 | 151 | 2.225 | NA | NA | NA | NA |
| Wanang | WS2 | Felling | Tropical Lowland Secondary | 1,988 | 0.45 | 88 | 172 | 3,328.60 | 1,791 | 167 | 982 | NA | NA | NA | NA |
| Wanang | WS3 | Felling | Tropical Lowland Secondary | 1,833 | 0.46 | 89 | 123 | 1,688.33 | 475 | 17 | 294 | NA | NA | NA | NA |
| Wanang | WS4 | Felling | Tropical Lowland Secondary | 1,484 | 0.58 | 87 | 132 | 2,355.16 | 2,605 | 58 | 1,293 | NA | NA | 1249 | 131 |
| Wanang | WS5 | Felling | Tropical Lowland Secondary | 785 | 0.51 | 89 | 45 | 1,767.06 | 2,432 | 163 | 1,565 | NA | NA | 1,615 | 186 |
| Wanang | WS6 | Felling | Tropical Lowland Secondary | 1,369 | 0.45 | 83 | 118 | 4,034.45 | 2,486 | 273 | 1,930 | NA | NA | NA | NA |
| Wanang | WS7 | Felling | Tropical Lowland Secondary | 907 | 0.57 | 81 | 45 | 2,999.24 | 1,191 | 166 | 1,655 | NA | NA | NA | NA |
| Wanang | WS8 | Felling | Tropical Lowland Secondary | 1,801 | 0.90 | 82 | 108 | 1,891.99 | 791 | 325 | 1,067 | NA | NA | NA | NA |
| Wanang | WS9 | Felling | Tropical Lowland Secondary | 1,699 | 0.97 | 88 | 101 | 1,886.50 | 2,458 | 270 | 2,282 | NA | NA | 1,188 | 132 |
| **Site** | **Plot** | **Method** | **Forest type** | **Area-based sampling effort**  **(man hours)** | **Resource-**  **Based sampling effort (man hours)** | **Canopy**  **sampled (%)** | **Stems**  **(DBH≥5cm)** | **Leaf area**  **(m^2^)** | **Caterpillars** | **Active**  **mines** | **Abandoned**  **mines** | **Galls** | **Spiders** | **Foraging**  **ants** | **Ant nests** |
| Wanang | WS10 | Felling | Tropical Lowland Secondary | 1,704 | 0.81 | 81 | 127 | 1,699.70 | 1,740 | 82 | 2,270 | NA | NA | 1,535 | 222 |
| Fort Sherman | Panama 1 | Crane | Tropical Lowland Primary | 2,698 | 1.21 | 87 | 95 | 2,237.41 | 1,341 | 1689 | 6577 | 193,215 | NA | NA | NA |
| Fort Sherman | Panama 2 | Crane | Tropical Lowland Primary | 2,110 | 1.17 | 79 | 86 | 1,808.31 | 275 | 327 | 4699 | 45,974 | NA | NA | NA |
