# Supplementary material for: Quantitative assessment of plant-arthropod interactions in forest canopies: A plot-based approach
Source: PLoS One. 2019 Oct 23;14(10):e0222119. doi: 10.1371/journal.pone.0222119 (PMC6808442; doi:10.1371/journal.pone.0222119)
Supplement: S3 Table — (DOCX) [file pone.0222119.s005.docx]

**Quantitative assessment of arthropod-plant interactions in forest canopies: a plot-based approach**

Martin Volf, Petr Klimeš, Greg Lamarre, Conor Redmond, Carlo L. Seifert, Tomokazu Abe, John Auga, Kristina Anderson-Teixeira, Yves Basset, Saul Beckett, Philip T. Butterill, Pavel Drozd, Erika Gonzalez-Akre, Ondřej Kaman, Naoto Kamata, Benita Laird-Hopkins, Martin Libra, Markus Manumbor, Scott E. Miller, Kenneth Molem, Ondřej Mottl, Masashi Murakami, Tatsuro Nakaji, Nichola S. Plowman, Petr Pyszko, Martin Šigut, Jan Šipoš, Robert Tropek, George Weiblen, and Vojtech Novotny

**S3 Table**. Monthly trends in abundance of caterpillars and active miners across the plots sampled for multiple months (Tomakomai, Lanzhot, Toms Brook, San Lorenzo, Wanang, Numba, Yawan). The table shows averages (± standard deviation) of caterpillar and active miner abundance per m^2^ of foliage encountered at individual days of sampling within given months.

| **Caterpillars** | **Tomakomai** | **Lanžhot** | **Toms Brook** | **Wanang**  **primary** | **Wanang**  **secondary** | **San Lorenzo** | **Numba primary+secondary** | **Yawan primary+secondary** |
| --- | --- | --- | --- | --- | --- | --- | --- | --- |
| January |  |  |  | 0.3±0.2 | 0.3±0.2 | 0.5±0.5 |  | 0.9±1.7 |
| February |  |  |  | 0.1±0.1 | 0.5±0.3 | 0.3±0.1 |  | 1.3±3.2 |
| March |  |  |  | 0.1±0.2 | 0.7±0.5 | 0.3±0.5 | 0.4±0.2 | 0.7±1.1 |
| April |  |  | 8.8±10.1 | 0.3±0.4 | 1.1±1.3 | 0.2±0.2 | 0.3±0.2 | 0.4±0.4 |
| May | 41.9±51.4 | 11.4±15.2 | 1.5±2.6 | 0.4±0.7 | 1.5±0.8 | 0.0±0.0 | 0.5±0.3 | 0.3±0.2 |
| June | 10.4±9.1 | 1.1±1.1 | 1.0±1.1 | 0.6±0.8 | 1.6±1.3 | 0.1±0.1 | 0.5±0.4 | 0.2±0.2 |
| July | 1.0±0.8 | 0.7±0.4 | 2.1±1.1 | 0.8±0.8 | 1.2±1.7 | 0.4±0.6 | 0.6±0.4 | 0.3±0.4 |
| August | 0.5±0.1 | 5.3±8.8 | 1.4±1.2 | 0.2±0.2 | 2.1±4.9 | 0.8±0.7 | 0.4±0.2 | 0.4±0.7 |
| September |  |  |  | 0.4±0.6 | 0.5±0.3 | 0.5±1.1 |  | 0.4±0.5 |
| October |  |  |  | 0.1±0.1 | 0.9±1.2 | 0.9±1.7 | 0.1±0.2 | 0.2±0.1 |
| November |  |  |  | 0.0±0.0 | 0.3±0.2 | 0.3±0.5 | 0.5±0.5 | 0.2±0.2 |
| December |  |  |  |  | 0.4±0.4 | 0.5±0.7 |  | 0.3±0.2 |
| **Miners** | **Tomakomai** | **Lanžhot** | **Toms Brook** | **Wanang**  **primary** | **Wanang**  **secondary** | **San Lorenzo** | **Numba**  **primary+secondary** | **Yawan**  **primary+secondary** |
| January |  |  |  | 0.00±0.00 | 0.01±0.01 | 0.53±1.68 |  | 0.03±0.04 |
| February |  |  |  | 0.01±0.01 | 0.04±0.03 | 0.74±0.98 |  | 0.05±0.04 |
| March |  |  |  | 0.05±0.05 | 0.09±0.12 | 0.58±1.05 | 0.03±0.05 | 0.10±0.16 |
| April |  |  | 0.03±0.06 | 0.05±0.06 | 0.03±0.03 | 0.07±0.14 | 0.02±0.02 | 0.04±0.06 |
| May | 0.77±2.9 | 0.11±0.25 | 0.09±0.15 | 0.14±0.34 | 0.13±0.10 | 0.13±0.14 | 0.02±0.04 | 0.14±0.19 |
| June | 0.33±0.46 | 0.33±0.41 | 0.32±0.27 | 0.09±0.12 | 0.11±0.15 | 0.06±0.15 | 0.03±0.05 | 0.02±0.02 |
| July | 0.32±0.26 | 0.08±0.09 | 0.92±1.05 | 0.05±0.10 | 0.09±0.10 | 0.21±0.44 | 0.04±0.05 | 0.02±0.03 |
| August | 0.93±0.98 | 0.09±0.19 | 0.86±0.74 | 0.06±0.06 | 0.05±0.07 | 0.05±0.11 | 0.00±0.00 | 0.07±0.09 |
| September |  |  |  | 0.02±0.03 | 0.03±0.07 | 1.39±5.58 |  | 0.04±0.04 |
| October |  |  |  | 0.04±0.06 | 0.04±0.07 | 0.63±1.93 | 0.01±0.03 | 0.09±0.09 |
| November |  |  |  | 0.05±0.00 | 0.11±0.08 | 0.15±0.20 | 0.00±0.00 | 0.05±0.06 |
| December |  |  |  |  | 0.06±0.08 | 0.08±0.21 |  | 0.01±0.01 |
|  |  |  |  |  |  |  |  |  |
|  |  |  |  |  |  |  |  |  |
