# Supplementary material for: Quantitative assessment of plant-arthropod interactions in forest canopies: A plot-based approach
Source: PLoS One. 2019 Oct 23;14(10):e0222119. doi: 10.1371/journal.pone.0222119 (PMC6808442; doi:10.1371/journal.pone.0222119)
Supplement: S4 Table — (DOCX) [file pone.0222119.s006.docx]

**Quantitative assessment of arthropod-plant interactions in forest canopies: a plot-based approach**

Martin Volf, Petr Klimeš, Greg Lamarre, Conor Redmond, Carlo L. Seifert, Tomokazu Abe, John Auga, Kristina Anderson-Teixeira, Yves Basset, Saul Beckett, Philip T. Butterill, Pavel Drozd, Erika Gonzalez-Akre, Ondřej Kaman, Naoto Kamata, Benita Laird-Hopkins, Martin Libra, Markus Manumbor, Scott E. Miller, Kenneth Molem, Ondřej Mottl, Masashi Murakami, Tatsuro Nakaji, Nichola S. Plowman, Petr Pyszko, Martin Šigut, Jan Šipoš, Robert Tropek, George Weiblen, and Vojtech Novotny

**S4 Table**. Variables with a significant effects on **Foliage accessibility**, **Area-based sampling effort,** and **Resource-based sampling effort** as selected by forward selection in linear mixed effect models. The best model explaining differences in **Foliage accessibility** included fixed effects of the forest type, used method, number of stems with DBH≥5cm, and sampled leaf area (m^2^) (χ^2^ (8) =64.02, p<0.0001). Percentage data on **Foliage accessibility** were arcsine transformed. The best model explaining differences in **Area-based sampling effort** included fixed effects of number of stems with DBH≥5cm, and forest type (χ^2^ (5) =95.24, p<0.0001). The best model explain differences in **Resource-based sampling effort** included fixed effects of number of stems with DBH≥5cm, sampled leaf area, and forest type (χ^2^ (6) =80.75, p<0.0001). Effort data were log-transformed. Site was used as random effect.

| **Foliage accessibility** |  |  |  |  |
| --- | --- | --- | --- | --- |
| **Fixed effects** | **Estimate** | **Std. Error** | **t value** | **AIC** |
| *Null model* |  |  |  | -114.50 |
| *Selected model* |  |  |  | -162.52 |
| (Intercept) | 0.9259 | 0.0290 | 31.94 |  |
| Forest type |  |  |  |  |
| *Tropical highland primary* | 0.0468 | 0.0318 | 1.47 |  |
| *Tropical highland secondary* | 0.1167 | 0.0307 | 3.80 |  |
| *Tropical lowland primary* | 0.0569 | 0.0323 | 1.76 |  |
| *Tropical lowland secondary* | 0.1257 | 0.0284 | 4.44 |  |
| Method |  |  |  |  |
| *Felling* | -0.0201 | 0.0307 | -0.66 |  |
| *Cherry-picker* | 0.2112 | 0.0438 | 4.82 |  |
| Number of stems with DBH≥5cm | 0.0008 | 0.0002 | 4.59 |  |
| Sampled leaf area | 0.00003 | 0.00001 | -3.30 |  |
| **Area-based sampling effort** |  |  |  |  |
| **Fixed effects** | **Estimate** | **Std. Error** | **t value** | **AIC** |
| *Null model* |  |  |  | 56.13 |
| *Selected model* |  |  |  | -29.11 |
| (Intercept) | 6.788 | 0.106 | 63.95 |  |
| Number of stems with DBH≥5cm | 0.007 | 0.001 | 13.11 |  |
| Forest type |  |  |  |  |
| *Tropical highland primary* | -0.294 | 0.165 | -1.78 |  |
| *Tropical highland secondary* | -0.770 | 0.168 | -4.58 |  |
| *Tropical lowland primary* | 0.199 | 0.166 | 1.20 |  |
| *Tropical lowland secondary* | -0.106 | 0.171 | -0.62 |  |
| **Resource-based sampling effort** |  |  |  |  |
| **Fixed effects** | **Estimate** | **Std. Error** | **t value** | **AIC** |
| *Null model* |  |  |  | -70.96 |
| *Selected model* |  |  |  | -139.71 |
| (Intercept) | 0.709 | 0.058 | 12.32 |  |
| Number of stems with DBH≥5cm | 0.002 | 0.000 | 10.42 |  |
| Sampled leaf area | -0.000 | 0.000 | -7.66 |  |
| Forest type |  |  |  |  |
| *Tropical highland primary* | -0.268 | 0.097 | -2.77 |  |
| *Tropical highland secondary* | -0.393 | 0.096 | -4.07 |  |
| *Tropical lowland primary* | -0.058 | 0.096 | -0.60 |  |
| *Tropical lowland secondary* | -0.108 | 0.096 | -1.13 |  |
