# Supplementary material for: Quantitative assessment of plant-arthropod interactions in forest canopies: A plot-based approach
Source: PLoS One. 2019 Oct 23;14(10):e0222119. doi: 10.1371/journal.pone.0222119 (PMC6808442; doi:10.1371/journal.pone.0222119)
Supplement: S5 Table — (DOCX) [file pone.0222119.s007.docx]

**Quantitative assessment of arthropod-plant interactions in forest canopies: a plot-based approach**

Martin Volf, Petr Klimeš, Greg Lamarre, Conor Redmond, Carlo L. Seifert, Tomokazu Abe, John Auga, Kristina Anderson-Teixeira, Yves Basset, Saul Beckett, Philip T. Butterill, Pavel Drozd, Erika Gonzalez-Akre, Ondřej Kaman, Naoto Kamata, Benita Laird-Hopkins, Martin Libra, Markus Manumbor, Scott E. Miller, Kenneth Molem, Ondřej Mottl, Masashi Murakami, Tatsuro Nakaji, Nichola S. Plowman, Petr Pyszko, Martin Šigut, Jan Šipoš, Robert Tropek, George Weiblen, and Vojtech Novotny

**S5 Table**. List of staff, interns, students, volunteers, and local assistants who helped with the sampling.

| **Site** | **First Name** | **Second Name** | **Role** |
| --- | --- | --- | --- |
| **Tomakomai** |  |  |  |
| Tomakomai | Haruka | Abe | Plant and Arthropod sampling |
| Tomakomai | Hiroaki | Fukushima | Plant and Arthropod sampling |
| Tomakomai | Tsutom | Hiura | Field management |
| Tomakomai | Utsugi | Jinbo | Lepidoptera identification |
| Tomakomai | Ryosuke | Kogo | Plant and Arthropod sampling; sample processing |
| Tomakomai | Rajesh | Kumar | Plant and Arthropod sampling, Lepidoptera identification |
| Tomakomai | Roll | Lilip | Plant and Arthropod sampling (in charge of miner sampling and processing) |
| Tomakomai | Jan | Macek | Insect identification |
| Tomakomai | Junichi | Yukawa | Gall identification |
| **Lanžhot** |  |  |  |
| Lanžhot | Denisa | Bazsoová | Plant and Arthropod sampling; Insect rearing |
| Lanžhot | Ondřej | Dorňák | Plant and Arthropod sampling; Insect rearing |
| Lanžhot | Jiří | Hodeček | Plant and Arthropod sampling |
| Lanžhot | David | Kaspřák | Plant and Arthropod sampling |
| Lanžhot | Markéta | Kirstová | Plant and Arthropod sampling; Insect rearing |
| Lanžhot | Nela | Kotásková | Sampling, sample sorting |
| Lanžhot | Jan | Macek | Insect identification |
| Lanžhot | David | Musiolek | Plant and Arthropod sampling |
| Lanžhot | Hana | Platková | Plant and Arthropod sampling |
| Lanžhot | Veronika | Plocková | Plant and Arthropod sampling; Insect rearing |
| Lanžhot | Aneta | Sajdok | Plant and Arthropod sampling |
| Lanžhot | Mark | Shaw | Insect identification |
| Lanžhot | Stefan | Schmidt | Curator of parasitoid specimens |
| Lanžhot | Alžběta | Suchánková | Plant and Arthropod sampling |
| Lanžhot | Michal | Zapletal | Insect identification |
| **Mikulčice** |  |  |  |
| Mikulcice | Jaroslav | Baloun | Plant and Arthropod sampling |
| Mikulcice | Lukáš | Čížek | Help with preparation of the sampling and management |
| Mikulcice | Jaroslav | Dlouhý | Plant and Arthropod sampling |
| Mikulcice | Nela | Kotásková | Plant and Arthropod sampling |
| Mikulcice | Kateřina | Kuřavová | Plant and Arthropod sampling |
| Mikulcice | Jan | Macek | Insect identification |
| Mikulcice | Ivan | Mikuláš | Arthropod sampling |
| Mikulcice | Ondřej | Šulák | Plant and Arthropod sampling |
| Mikulcice | Štěpán | Vodka | Plant and Arthropod sampling |
| **Site** | **First Name** | **Second Name** | **Role** |
| Mikulcice | Jan | Vrána | Arthropod sampling, in charge of ant sampling |
| Mikulcice | Michal | Zapletal | Insect identification |
| Mikulcice | Tomáš | Zítek | Plant and Arthropod sampling |
| **Front Royal** |  |  |  |
| Front Royal | Thomas | Blair | Plant and Arthropod sampling |
| Front Royal | Grace | Carscallen | Plant and Arthropod sampling, in charge of ant sampling in 2016 |
| Front Royal | Maria Eugenia | Losada | Plant and Arthropod sampling, in charge of leaf miner-gall in 2016 |
| Front Royal | Inga | Freiberga | Gall sample processing and dissection |
| Front Royal | Aaron | Goodman | Sampling, in charge of spider sampling and identification in 2017 |
| Front Royal | Geoffrey | Nichols | Plant and Arthropod sampling, in charge of leaf miner-gall in 2017 |
| Front Royal | Margaret | Rosati | Logistic support |
| Front Royal | Matthias | Weiss | Gall sample processing and dissection |
| Front Royal | Kate | Aldrich | Plant and Arthropod sampling |
| Front Royal | Clayton | Hatcher | Plant and Arthropod sampling |
| Front Royal | Shelby | Abbott | Plant and Arthropod sampling |
| Front Royal | Meghan | Melberg | Plant and Arthropod sampling |
| Front Royal | Amanda | Gambale | Plant and Arthropod sampling |
| **San Lorenzo** |  |  |  |
| San Lorenzo | John | Auga | Plant and Arthropod sampling |
| San Lorenzo | Stefan | Curtis | Plant and Arthropod sampling, leader of the climbing team |
| San Lorenzo | Ondřej | Dorňák | Plant and Arthropod sampling |
| San Lorenzo | Inga | Freiberga | Gall sample processing and dissection |
| San Lorenzo | Domminik | Rabl | Plant and Arthropod sampling |
| San Lorenzo | Mariam | Trejos | Plant and Arthropod sampling |
| San Lorenzo | Matthias | Weiss | Gall sample processing and dissection |
| San Lorenzo | Joachim | Yalang | Plant and Arthropod sampling |
| San Lorenzo | Inga | Freiberga | Gall sample processing and dissection |
| San Lorenzo | Matthias | Weiss | Gall sample processing and dissection |
| **Wanang** |  |  |  |
| Wanang | Darren | Bito | Research Supervisor with a focus on parasitoids |
| Wanang | Erik | Brus | Arthropod sampling |
| Wanang | Kipiro | Damas | Data cleaning/Identifications |
| Wanang | Jan | Hrček | Research Supervisor with a focus on parasitoids |
| Wanang | Sentiko | Ibalim | Arthropod sampling |
| Wanang | Cliffson | Idigel | Arthropod sampling |
| Wanang | Bruce | Isua | Botany leader |
| Wanang | Robin | Kalwa | Plant sampling/vouchering |
| Wanang | Martin | Keltim | Arthropod sampling |
| Wanang | Andrew | Kinibel | Arthropod sampling |
| Wanang | Joseph | Kua | Arthropod sampling |
| Wanang | Roll | Lilip | Arthropod sampling |
| Wanang | Martin | Mogia | Arthropod sampling |
| Wanang | Kenneth | Molem | Botany leader |
| Wanang | Rebecca | Montgomery | Herbivory measures protocol |
| Wanang | Aloysius | Posman | Arthropod sampling |
| Wanang | Maling | Rimandai | Arthropod sampling |
| **Site** | **First Name** | **Second Name** | **Role** |
| Wanang | Steven | Sau | Arthropod sampling |
| Wanang | Gibson | Sosanika | Plant sampling/vouchering |
| Wanang | Elvis | Tamtiai | Arthropod sampling |
| Wanang | Tim | Whitfeld | Data cleaning/Identifications |
| **Numba** |  |  |  |
| Numba | Kenneth | Benedict | Field lab work, Sample processing (botany) |
| Numba | Bradley | Gewa | Field lab work, Plant and Arthropod sampling, sample processing |
| Numba | Amelia | Hood | Field lab work, Sample processing |
| Numba | Frank | Jurgen | Field lab work |
| Numba | Graham | Kaina | Field lab work, Plant and Arthropod sampling, sample processing |
| Numba | Martin | Keltim | Plant and Arthropod sampling, sample processing |
| Numba | Andrew | Kinibel | Plant and Arthropod sampling, sample processing |
| Numba | Nancy | Labun | Sample processing (plants) |
| Numba | Roll | Lilip | Field lab work |
| Numba | Grace | Luke | Sample processing (arthropods) |
| Numba | Gibson | Maiah | Arthropod sampling |
| Numba | Gibson | Mayiah | Plant and Arthropod sampling, sample processing |
| Numba | Frank | Philip | Plant and Arthropod sampling, sample processing |
| Numba | Steven | sau | Field lab work |
| **Yawan** |  |  |  |
| Yawan | Beneth | Ara | Plant and Arthropod sampling, sample processing |
| Yawan | Semcars | Ara | Plant and Arthropod sampling, sample processing |
| Yawan | Steven | Ganya | Plant and Arthropod sampling, sample processing |
| Yawan | Bradley | Gewa | Field lab work |
| Yawan | Hendry | Ginsongne | Plant and Arthropod sampling, sample processing |
| Yawan | Menos | Ginsongne | Plant and Arthropod sampling, sample processing |
| Yawan | Henson | Gomes | Plant and Arthropod sampling, sample processing |
| Yawan | Wotimo | Guboingnuc | Plant and Arthropod sampling, sample processing |
| Yawan | Bridget | Henning | Research supervisor; Sample collection |
| Yawan | Amelia | Hood | Sample processing |
| Yawan | Cliffson | Idigel | Field lab work |
| Yawan | Brus | Isua | Field lab work |
| Yawan | Tonsep | Joseph | Management of field assistants, field management |
| Yawan | Samuel | Joseph | Plant and Arthropod sampling, sample processing |
| Yawan | Martin | Keltim | Field lab work |
| Yawan | Barnabas | Kombe | Plant and Arthropod sampling, sample processing |
| Yawan | Joseph | Kua | Field lab work |
| Yawan | Oberth | Kui | Plant and Arthropod sampling, sample processing |
| Yawan | Roll | Lilip | Field lab work |
| Yawan | Bill | Lodi | Plant and Arthropod sampling, sample processing |
| Yawan | Max | Manaono | Plant and Arthropod sampling, sample processing |
| Yawan | Kenny | Mangirai | Plant and Arthropod sampling, sample processing |
| Yawan | Markus | Manumbor | Field lab work, Team leader |
| Yawan | Gibson | Mayiah | Field lab work |
| Yawan | Martin | Mogia | Field lab work |
| Yawan | Robert | Mongo | Plant and Arthropod sampling, sample processing |
| **Site** | **First Name** | **Second Name** | **Role** |
| Yawan | Jim | Nasing | Plant and Arthropod sampling, sample processing |
| Yawan | Aikson | Nea | Plant and Arthropod sampling, sample processing |
| Yawan | Namuce | Nongi | Plant and Arthropod sampling, sample processing |
| Yawan | Walindong | Nonong | Plant and Arthropod sampling, sample processing |
| Yawan | Frank | Philip | Plant and Arthropod sampling, sample processing |
| Yawan | Maling | Rimandai | Field lab work |
| Yawan | Sawaing | Sorong | Plant and Arthropod sampling, sample processing |
| Yawan | Wrefords | Sorong | Plant and Arthropod sampling, sample processing |
| Yawan | Gibson | Sosanika | Field lab work |
| Yawan | Elvis | Tamtiai | Field lab work |
| Yawan | Alu | Tonsep | Plant and Arthropod sampling, sample processing |
| Yawan | Sesilin | Tonsep | Plant and Arthropod sampling, sample processing |
| Yawan | Maxon | Tonseph | Plant and Arthropod sampling, sample processing |
| Yawan | Salape | Tulai | Field lab work |
| Yawan | Joseph | Valeba | Field lab work |
| Yawan | Mangan | Witwit | Plant and Arthropod sampling, sample processing |
