# Supplementary material for: Quantitative assessment of plant-arthropod interactions in forest canopies: A plot-based approach
Source: PLoS One. 2019 Oct 23;14(10):e0222119. doi: 10.1371/journal.pone.0222119 (PMC6808442; doi:10.1371/journal.pone.0222119)
Supplement: S1 Appendix — (PDF) [file pone.0222119.s008.pdf]

Quantitative assessment of arthropod-plant interactions in forest canopies: a plot-based approach

PLOS ONE

Martin Volf[1,2*], Petr Klimeš[1], Greg P.A. Lamarre[1,3], Conor M. Redmond[1,3], Carlo L. Seifert[1,3], Tomokazu Abe[4], John Auga[5], Kristina Anderson-Teixeira[6,7], Yves Basset[1,3,7,8], Saul Beckett[7], Philip T. Butterill[1,3], Pavel Drozd[9], Erika Gonzalez-Akre[6], Ondřej Kaman[1,3], Naoto Kamata[10], Benita Laird-Hopkins[11], Martin Libra[1,3], Markus Manumbor[5], Scott E. Miller[12], Kenneth Molem[5], Ondřej Mottl[1,3], Masashi Murakami[4], Tatsuro Nakaji[13], Nichola S. Plowman[1,3], Petr Pyszko[9], Martin Šigut[9], Jan Šipoš[14,15], Robert Tropek[1,16], George D. Weiblen[17], and Vojtech Novotny[1,3]

[1]Biology Centre of the Czech Academy of Sciences, Ceske Budejovice, CZ; [2]German Centre for Integrative Biodiversity Research (iDiv) Halle-Jena-Leipzig, Leipzig, DE; [3]Faculty of Science, University of South Bohemia, Ceske Budejovice, CZ; [4]Faculty of Science, Chiba University, Chiba, JP; [5]New Guinea Binatang Research Center, Madang, PG; [6]Smithsonian Conservation Biology Institute, Front Royal, VA, US; [7]Smithsonian Tropical Research Institute, Ancon, PA; [8]Maestria de Entomologia, Universidad de Panama, Panama City, PA; [9]Faculty of Science, University of Ostrava, Ostrava, CZ; [10]Graduate School of Agricultural and Life Sciences, The University of Tokyo, Tokyo, JP; [11]School of Biological Sciences, University of Bristol, Bristol, UK; [12]National Museum of Natural History, Smithsonian Institution, Washington, DC, US; [13]Tomakomai Experimental Forest, Hokkaido University, Tomakomai, JP; [14]Institute of Botany, Czech Academy of Sciences, Brno, CZ; [15]Department of Zoology, Fisheries, Hydrobiology and Apiculture, Mendel University in Brno, Brno, CZ; [16]Department of Ecology, Faculty of Science, Charles University, Prague, CZ; [17]Bell Museum and Department of Plant & Microbial Biology, University of Minnesota, Saint Paul, MN, US

*corresponding author: Martin Volf, volf@entu.cas

S1 Appendix. Sampling protocols

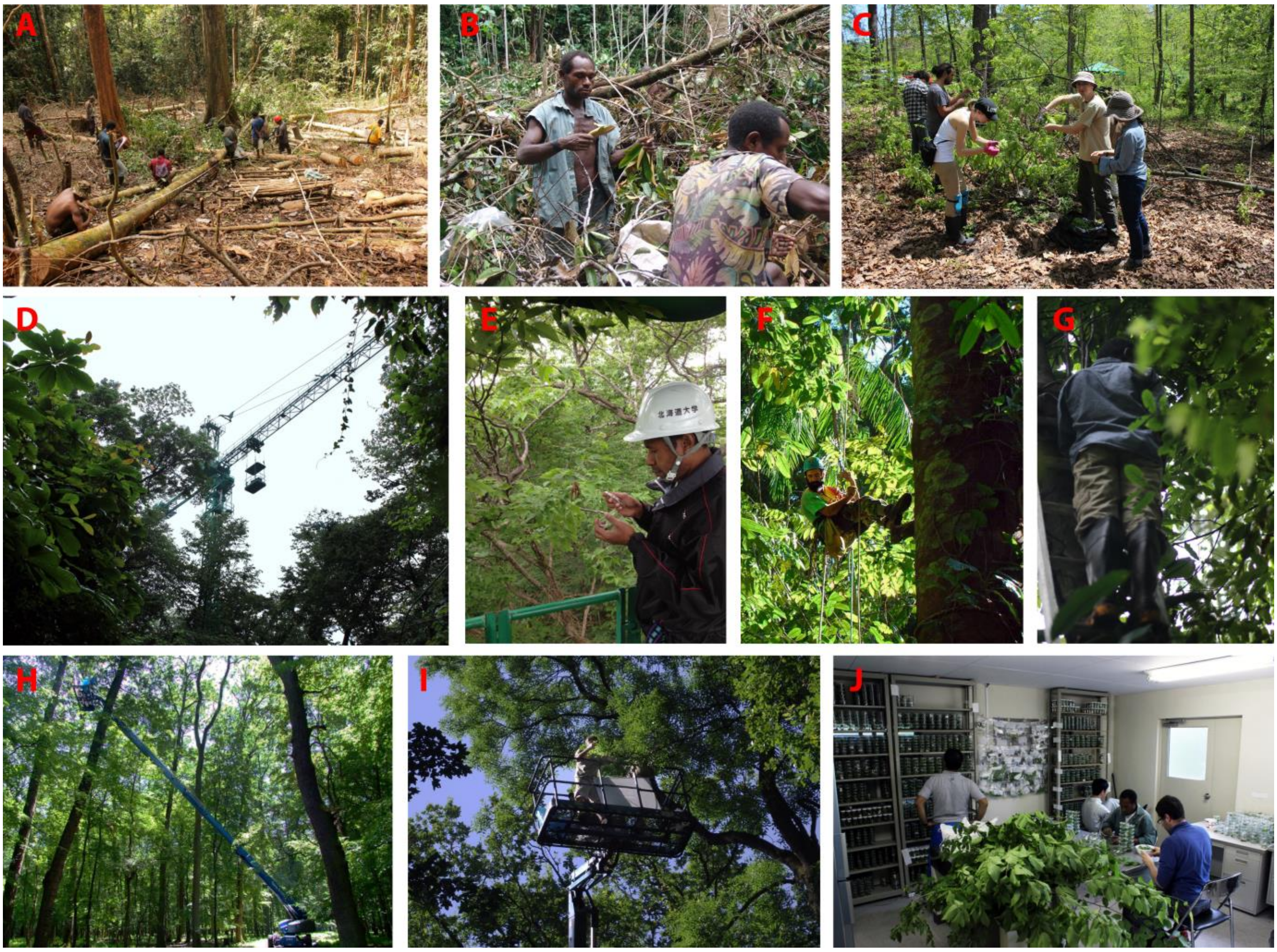

Quantitative assessment of arthropod-plant interactions in forest canopies: a plot-based approach

Content:

1.0 Setting up a 0.1ha plot……3

2.0 Arthropod sampling……4

2.1 Arthropod sampling from felled trees……4

2.2 Arthropod sampling from cranes and cherry-pickers……8

3.0 Leaf area estimates……12

3.1 Leaf area estimates for felled trees……12

3.2 Leaf area estimates for trees sampled from cranes and cherry-pickers……13

4.0 Sample processing and insect rearing……15

References……22

Example Plant Form (felling)……23

Example Plant Form (canopy cranes and cherry-pickers)……25

Example Ant Form……27

Example Ant Labels……28

1.0 Setting up a 0.1 ha plot

We propose a **standardized protocol for sampling 0.1ha forest plots to quantify interaction networks of canopy arthropods**. The choice of forest area depends on the characteristics of the forest structure and composition meeting all suitable requirements for your project and research questions. Allocate the necessary time to explore and find a suitable forest site. In particular, you should base your decision on the presence of invasive species, topography, and access to the plot (important for the removal of felled trees or a for cherry-picker access). Before you start your project, always inform yourself on all safety instructions applicable to working in the field. These are not included in this protocol. Anyone conducting the sampling is responsible for obtaining the safety instructions elsewhere and following them.

1. Select a plot, which represents a 0.1 ha with a structure and a species composition typical for the local forests. Avoid forest edges, gaps, heavily disturbed areas, sloped terrain, and plantations.

2. Set up the corner points of the plot and take GPS coordinates for reference. Use a measuring tape or a laser range finder to measure the distance between points. Use a compass to measure the angles between the corner points in order to set up the plot in the desired shape. You can use a standard or electronic compass for this. Artillery compasses, specifically designed for taking azimuth angles, are usually a good option.

3. Mark the trees with DBH ≥ 5 cm with labels and identify them to species level (the identifications can be improved once the canopy is accessed). Mark only the trees which are rooted in the plot. If the border of the plot goes through tree trunk, include the tree in the plot only if more than 50% of the trunk mass at breast height is within the plot perimeter.

4. Record the position of all trees within the plot. First, select a “ZERO” point within the plot from which you can see all the trees. Clear the understory vegetation to improve the visibility if necessary. You can also use brightly coloured marks (or somebody in bright clothing standing next to the trees) to further increase the visibility of individual trees. Then record the azimuth angle (using a compass) and distance (using a measuring tape or a laser range finder) of individual trees from this point. These can be later easily transformed into x and y coordinates.

5. Optional. If visibility cannot be improved by removing some of the understory vegetation, divide the plot into a grid (Fig. P1). Measure the position of their corner points and all the trees in individual sub-plots as described above. If this method is not possible, you can also take GPS coordinates of individual trees. However, this can be rather inaccurate compared to the previous method depending on the precision of your GPS.

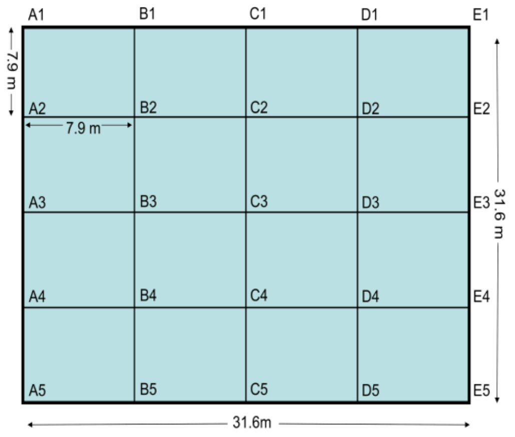

Figure P1. Example of a 0. 1ha plot divided into a grid with several reference points (A1-E5). Having such a grid improves accuracy of setting up the plot in densely vegetated sites.

2.0 Arthropod sampling

In temperate (and other seasonal) forests, sampling needs to be spread seasonally within each target tree species to capture the seasonal variability in associated arthropod communities. Create a sampling plan according to the phenology in the focal region (e.g. spread your sampling across both the spring and summer peak of arthropod abundance if such peaks are typical). Avoid sampling all conspecific trees in one part of the season if possible. Spreading sampling across the season may be problematic in the case of singleton tree species. Some methods, such as forest felling, provide limited flexibility for seasonal targeting of singleton tree species as trees cannot be resampled and the data thus represent a single time-point. On the other hand, sampling from cranes or cherry-pickers provides more flexibility. If there are any singleton tree species in your crane or cherry-picker plot, sample half of their canopy during the (spring) peak of arthropod abundance, while the second half can be sampled later in the season.

2.1 Arthropod sampling from felled trees

General notes

First, prepare a sampling plan to establish an ideal sequential order from which trees should be felled. Make sure individual tree species have a similar proportion of individuals sampled in different parts of the season. Clear the understorey. Start with felling small trees. Once enough small trees are gone and a sufficient space is opened, proceed with the larger trees. Always start with trees that are least likely to fall in a manner which may destroy other trees. This will minimize disturbance to the plot.

Trees should be felled one at a time. It is necessary to finish sampling on the same day as the tree was felled. All arthropods should be sampled as quickly as possible. This will prevent them from escaping or being predated.

Sampling should be done only during the day and when the leaves are not too wet. Avoid sampling in heavy rain, or directly after heavy rain (give the leaves some time to dry). Also avoid sampling during strong wind.

Divide sampling responsibilities within your team. If the size of your team allows, form sorting and sampling teams. Forming a sorting team, which will start pre-sorting samples in the field, will speed-up the final sorting in the lab; 2-3 team members are usually enough for pre-sorting.

There should be always skilled researchers and entomologists present in the field supervising the sampling and sample processing. Other team members should specialize primarily on a single arthropod group (leaf-chewing larvae, miners, or galls etc.) and be trained in the identification of their focal arthropod taxon prior to sampling. These specialized team members then can help other team members with assigning preliminary morphospecies and assist the skilled researcher with final morphotyping (see below).

Sampling steps

1. Select the tree to be felled according to your sampling plan. Measure its DBH (at 1.3 m).

2. Fell the tree

3. Measure its total height, trunk height, and canopy width. Trunk height is measured to the first major branch. Canopy width is measured at the widest point of the canopy. Record this into 'Plant Form'.

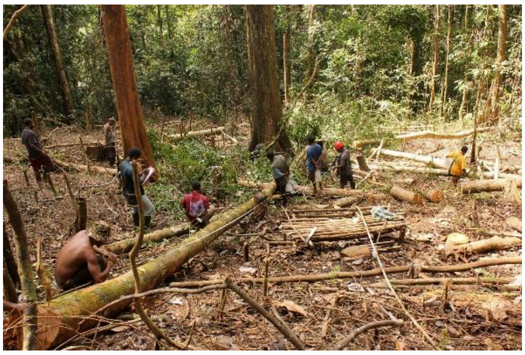

Figure P2. Measuring a felled tree in Numba.

4. Record whether the leaves are mature or young (developing). In temperate forests, almost all leaves on a tree will be either mature or

young at the time of sampling. In the tropics, this may not be the case so record mature and young leaves separately (see below in *Leaf area estimates*).

5. Sample the focal arthropod groups systematically by a manual search (see details on sampling of individual arthropod groups below). Hand the samples to the sorting team (if there is any) regularly during sampling. This is a much more efficient strategy than passing the samples all at once after the sampling is finished.

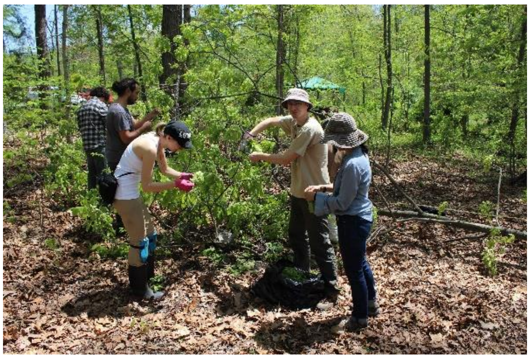

Figure P3. Sampling arthropods from a small felled tree in Toms Brook.

6. After the sampling, estimate what percentage of the foliage was sampled for arthropods (since part of the canopy usually gets destroyed during felling and you cannot sample herbivores from it). Record in 'Plant Form'. Estimates should be done by two trained persons independently and the mean estimated value should be used. This provides more accurate results.

These are the following arthropod groups to be sampled:

Leaf-chewing insect larvae

Search for all free-living and semi-concealed larvae. Check all rolled, tied, or folded leaves. Sample each larva in a separate rearing container. Gregarious larvae can be placed into a single large container, record their quantity. Containers should be available in various sizes suitable for larvae of different sizes. Provide a reasonable amount of leaves based on the size of the larva. The leaves should be of the same age the larva was sampled from (i.e. mature or young). Provide the larva with both young and mature leaves if you are not sure what leaves the larva was feeding on. Do not overfill the container with leaf material and keep it in the shade.

Miners

Sample all active and record all abandoned mines. When sampling mines try to assign them to preliminary morphospecies based on their shape, size, and position on the leaf. Mainly, separate blotch and serpentine mines. Keep your preliminary morphospecies in separate bags. Your preliminary morphotyping will be later corrected by an expert during final processing, but doing preliminary morphotyping and keeping your preliminary morphospecies in separate bags will speed up the final sorting.

Active mines

- Do not sample just the leaf with the mine. Mines will last longer if the leaf is attached to a twig with a couple of other leaves (but make sure that no other mine morphospecies are on the same leaves).
- Put all active mines from one morphospecies in one bag (they will be separated later). If you are not sure whether the mine is active or abandoned, sample it (it can be checked in detail later) and put it among other active mines from the respective morphospecies. Do not overfill the bag with leaf material and keep it in a shade.
- Sample up to ca 100 active mines per morphospecies only (50 will be used for rearing, 10 will be put in ethanol, and the rest will serve as a reserve in case some mines you sampled are inactive).
- The mines exceeding 100 can be simply counted (or their abundance can be estimated if there are many of them; see below). Record the number exceeding 100 into your notebook and report it to the sorting team after sampling. Always confirm with the expert assigning mines to final morphospecies that these mines are truly from a single morphospecies before you stop sampling them.

Abandoned mines

- Usually, you do not have to sample all abandoned mines. Just count their number or estimate their abundance visually in the event where there are too many of them (see below; but always confirm with the expert assigning mines to final morphospecies that these mines are truly from a single morphospecies). Record their number into your notebook and report it to the sorting team after finishing the sampling.
- Sample abandoned mines only if you do not have any active mine of that morphospecies available or assigning to clear morphospecies is problematic.

Gallers

- Sample all galls on all above-ground plant parts. When sampling galls, try to assign them to preliminary morphospecies. Mainly, focus on the plant part galled and shape of the gall. Your preliminary morphotyping will be later corrected by the expert doing the final processing, but doing preliminary morphotyping and keeping your preliminary morphospecies in separate bags will speed up the final sorting. It can be hard to distinguish arthropod and fungal galls. If unsure, sample all galls. Fungal galls can be identified in the laboratory and later removed from the analysis.
- Sample galled plant parts by detaching from the tree. If the galls are to be reared, and are in low numbers, galls will last longer if the plant part is attached to a twig with a couple of leaves. Otherwise, sample only the galled plant parts, preferably with active (inhabited) galls.
- Put different morphospecies in separate collecting bags. Do not overfill the bags and keep them in the shade.
- Sample enough galled material for each morphospecies to provide healthy quantities for rearing and dissection. What is considered a "healthy quantity" is dependent on the available resources (space, manpower, etc.) for rearing and dissecting, and the size of the galls. The more material reared and dissected, the better the chances of yielding insightful information to aid the species concept. Therefore, it would be ideal to rear at least 10 galled parts and retain at least 10 galls for dissection, per morphotype.
- Unsampled galls can be counted (or their abundance estimated if there are many of them; but always confirm with the expert assigning galls to final morphospecies that these galls are truly from a single morphospecies). Record the unsampled number into your notebook and report it to the sorting team after sampling.

Abundance estimates for very abundant mines and galls

Some abandoned leaf mines or gall morphospecies can be very abundant, which means counting them may take an excessive time investment. Instead of counting them individually, you can estimate their abundance in such cases. Mine and gall density can sometimes largely differ among various parts of the canopy. It is thus necessary to do the estimates repeatedly in various parts of the canopy.

- Select a reasonably large branch (ca 100-500 leaves) and count number of leaves and number of mines or galls on this branch. Divide their number by the number of leaves to calculate mine or gall per leaf average for this branch. Repeat this procedure at various parts of the canopy (at least three in the case of smaller trees and at least five in the case of larger trees). Use the averages to calculate a mean mine or gall density per individual leaf. Record this value. This can be used for estimating total mine or gall density once the total number of leaves is calculated.
- Some mite galls can be highly abundant (hundreds of galls per leaf). In such a case, pick only 20 leaves in random and calculate gall/leaf average. Repeat this procedure at various parts of the canopy (at least three in the case of smaller trees and at least five in the case of larger trees). Use the averages to calculate final mean gall density per individual leaf. Record this value. This can be used for estimating total gall density once the total number of leaves is calculated. Use this approach scarcely and only when really needed; e.g. in cases when more than 50% of leaves are galled.

- It is always better if the estimates are done by two specially trained persons using the mean estimate as a final value as it may provide more accurate results.

Spiders

Sample spiders into a vial with ethanol. All spiders from one tree can go into one vial but do not overfill it. Divide the spiders into more vials as needed to ensure a good proportion between ethanol and the sampled individuals. Similarly to ants (see below), sample spiders also from all lianas and epiphytes associated with the sampled tree.

Ants

Three people should be collecting ants (1 ant-trained staff member supervising 2 assistants) in tropical areas. In the temperate zone, where vegetation is less complex, two persons are enough. Sampling of foragers is done first immediately after felling. This helps to avoid contamination by ants invading the felled tree from the ground. After sampling for foragers is complete, collection continues with a search for individual nests.

Starting from the base of the tree (trunk) towards its crown, search carefully for any ants present on the fallen tree, especially those:

- foraging on the tree
- nesting on the leaves (silk or carton nest, weaved leaf nests etc.)
- living on and inside of the branches or twigs (Fig. P4)
- in the tree cavities
- under the bark
- under the lianas attached to the tree
- in the epiphytes on the tree, especially in the soil around their roots
- in any other suitable place where ants can occur

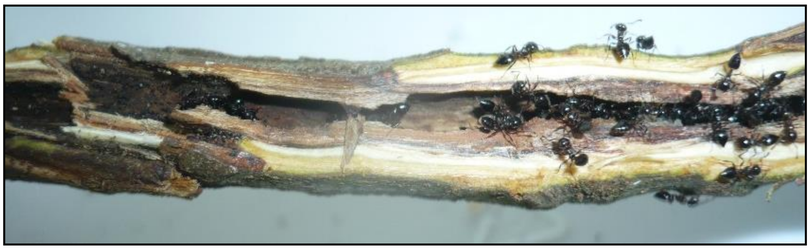

Figure P4. Ants often nest inside twigs and other host tree tissues. Do not forget to inspect even small twigs for ant nests. Use an axe or a chainsaw to cut open trunk and branches cavities for nests.

- We record several extra pieces of information for ants (such as their position on the tree, nest type etc.). This information should be recorded immediately after sampling, and recorded on both the labels and the 'Ant protocol' (see the example below). Do not wait till final processing to record this information.
- For all foragers, record their position on the tree – T (trunk below the branches) or C (crown – branches). All foraging ants (without a known nest) from one tree and similar height (T vs. C) can go together in one vial – this vial can contain a mix of different species If there is more than one vial with ants, mark each collection with a number: 1, 2, 3…
- For all nests, record their position (crown vs. trunk plus the vertical height above ground in meters), nest site type, and nest dimensions. Estimate the number of ant individuals in the nest. Record this information immediately after finding the nest. The examples of nest site types are listed below.
- Take vouchers of ant nests for photography (see *Sample processing and insect rearing*).
- Smaller colonies should be collected whole – including eggs, larvae and pupas and allates. Information as to whether the colony was collected as a whole is marked in the protocol and on labels.

- If the colony is too big (thousands of individuals), collect just part of it (20-50 individuals typically). Always try to sample all castes you can find as well as immature stages. Vials should be filled no more than halfway (1/2) with insects, the upper half should contain only ethanol to permit later molecular analysis (e.g. species barcoding). Use 2 ml vials for small samples. Use larger (e.g. 8 ml) vials for large bodied ants or larger colony samples.
- Ants from one colony (nest) should always be collected into one vial. They can be split in two, if there are too many ants for one vial – especially for big ants. In this case, each vial has to get its own label but with duplicated information. Don't mix ants from different colonies.
- Record if the host trees, or the ant-associated epiphytes, are myrmecophytes. Note if the plant contained ant domatia or nectaries (see an example of 'Ant protocol' below). Assigning plants as myrmecophytes or non-myrmecophytes can be difficult in tropical regions with poorly known flora and ant associations. Therefore, it is always crucial to record all the additional information as described above. The information on the location of the nest in dead or living tissue and trunk or branches can be especially helpful.

Nest types (write on the back side of your labels):
i) under the bark, ii) in hollow trunk, iii) in hollow live branch (= branch more than 5cm in diameter), iv) in hollow live twig (= branch less than 5cm in diameter, v) in hollow dead/dry branch, vi) in hollow dead/dry twig, vii) in hollow liana, vii) in/under epiphyte roots (or aerial soil), viii) inside of myrmecophytic epiphyte, ix) under liana, x) silk/carton nest on leaf, xi) carton nest on trunk/branch, xii) no nest (used for foraging individuals).

2.2 Arthropod sampling from cranes and cherry-pickers

General notes

First prepare a sampling plan, outlining the order in which the trees should be sampled. The primary aim here should be to account for seasonality. If the herbivore composition changes with the seasonal, ensure that you distribute sampling of conspecific tree individuals across the season. Avoid sampling all conspecific tree individuals in one part of season. If there are singleton tree species in your plot, sample 50% of their canopy in early season and the other 50% in later season.

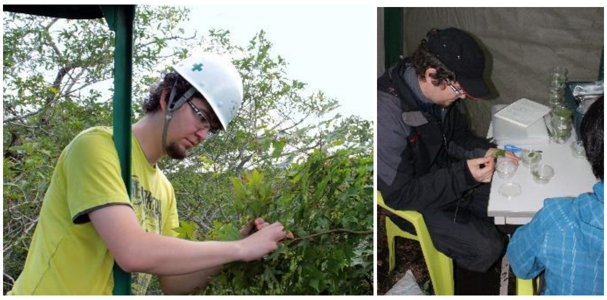

Figure P5. Canopy sampling and ground sample sorting in Tomakomai.

Sampling should be done only during the day and when the leaves are not very wet. Avoid sampling in heavy rain, or directly after heavy rain (give the leaves some time to dry). Also avoid sampling during windy weather.

Divide sampling responsibilities within your team. If the size of your team allows, form sorting and sampling teams. Forming a sorting team, which will start pre-sorting samples in the field, will speed-up the final sorting in the lab; 2-3 team members are usually enough for the pre-sorting. Ideally, there should be a skilled researcher present in both teams.

Sampling steps

1. Follow your sampling plan to select the tree to be sampled.

2. Measure the tree. First, measure the DBH (at 1.3 m). Then measure total height, trunk height, canopy width using a laser range finder. Trunk height is measured to the first major branch. Canopy width is measured at the widest point of the canopy. Record these values in 'Plant Form'.

3. Record whether the leaves are mature or young (developing). In temperate forest, almost all leaves on a tree will be either mature or young at the time of sampling. In the tropics, this may not be the case so record mature and young leaves separately (see *Leaf area estimates* for more details).

4. Sample the focal arthropod groups. First, use a beating net to obtain free living arthropods. Second, do a manual search to obtain remaining caterpillars, ants and spiders and also herbivores concealed in rolled or tied leaves, galls and mines. Hand the insect samples to the ground team during the sampling regularly. This is much more efficient strategy than passing the samples all at once after finishing sampling.

5. After the sampling, estimate what percentage of the foliage was sampled for arthropods. Record it into the 'Plant Form'. This should be done by the canopy team. Estimates should be done by two trained persons independently and the mean estimated value should be used. This provides more accurate results.

6. Record the number of leaves inspected for arthropods (see the instructions below in *Leaf area estimates*). Canopy team should report this value to the ground team immediately after sampling.

Sampling low accessibility parts of the canopy

Some parts of the canopy (usually understory trees or lower branches of large trees) can be inaccessible from cranes or cherry-pickers. In such cases, you can use sampling from the ground, from ladders, or by climbing. If climbing is necessary, it usually requires forming a specialized climbing team consisting of 1-2 specially trained team members.

- Trees with height of 2-3 m can usually be sampled directly from the ground. Be careful not to break any branches or the trunk. Rather than bending such a tree by a brutal force, use a ladder.
- We used "A" shaped step ladders for sampling up to 3-5 m above ground (depending on the type, its stability, and terrain). In the case of large trees with sufficient trunk diameter, extension ladders fixed to the trunk can be also used for reaching similar heights. Always make sure the ladder is stable. During our sampling, the person on the ladder was always assisted by at least one person on the ground. We avoided using this type of ladder on sloped terrain.
- For sampling at greater heights or on sloped terrain, modular ladder poles are more efficient and stable. We used ladder poles for sampling at up to 8 m above ground. But note that this may differ depending on the type you use and its maximum load. The ladder poles should be ideally equipped with a steel fork at the basis that ensures good stability of the pole in the ground. We secured the ladder pole to the trunk of the tree with harnesses to prevent it from slipping. The person on the ladder was always assisted by at least one person on the ground.
- Trees even higher above ground, which are inaccessible from cranes or cherry-pickers, can be sampled by climbing. Descending from the gondola can ensure that even the terminal branches can be reached. But this method is usually time consuming. Also, it can only be carried out by a skilled person with proper training.
- Untrained or inexperienced team members should never sample from ladders or climb the trees.
- Always read and carefully follow safety instructions which may apply to working in the field, to working at heights, to working from ladders, or to climbing. This protocol cannot be used as a source of such information. You must obtain all the safety regulations from elsewhere and follow them.

These are the following arthropod groups to be sampled:

Leaf-chewing insect larvae

Collect all leaf-chewing larvae from the beating net. Then search for all free-living and semi-concealed larvae. Check all rolled, tied, or folded leaves. Sample each larva in a separate rearing container. Gregarious larvae can be sampled into a single large container, record their quantity. Containers should be available in various sizes suitable for larvae of different sizes. Provide a reasonable amount of leaves based on the size of the larva. The leaves provided should be of the same age as those the larva was sampled from (i.e. mature or young). Provide the larva with both young and mature leaves if you are not sure what leaves the larva was feeding on. Do not overfill the container with leaf material and keep it in a shade.

Miners

Sample all active and record all abandoned mines. When sampling mines try to assign them to preliminary morphospecies based on their shape, size, and position on the leaf. Specifically, separate blotch and serpentine mines. Keep your preliminary morphospecies in separate bags. Your preliminary morphotyping will be later corrected by an expert during final processing, but doing preliminary morphotyping and keeping your preliminary morphospecies in separate bags will speed up the final sorting.

Active mines:

- Do not sample just the leaf with the mine. Mines will last longer if the leaf is attached to a twig with a couple of other leaves (but make sure that no other mine morphospecies are on the same leaves).
- Put all active mines from one morphospecies in one bag (they will be separated later). If you are not sure whether the mine is active or abandoned, sample it (it can be checked in detail later) and put it among other active mines from the respective morphospecies. Do not overfill the bags with leaf material and keep them in a shade.
- Sample up to ca 100 of active mines per morphospecies only (50 will be used for rearing, 10 will be put in ethanol, and the rest will serve as a reserve in case some mines you had sampled are inactive).
- The mines exceeding 100 can be simply counted (or their abundance can be estimated if there are many of them). Record the number exceeding 100 into your notebook and report it to the sorting team after sampling. Always confirm with the expert assigning mines to final morphospecies that these mines are truly from a single morphospecies before you stop sampling them.

Abandoned mines:

- Usually, you do not have to sample all abandoned mines. Just count their number or estimate their abundance visually in the event where there are too many of them (see below; but always confirm with the expert assigning mines to final morphospecies that these mines are truly from a single morphospecies). Record their number into your notebook and report it to the sorting team after finishing the sampling.
- Sample abandoned mines only if you do not have any active mine of that morphospecies available or assigning to clear morphospecies is problematic.

Gallers

- Sample all galls on all above-ground plant parts. When sampling galls, try to assign them to preliminary morphospecies. Specifically, focus on the plant part galled and shape of the gall. Your preliminary morphotyping will be later corrected by an expert during the final processing, but doing preliminary morphotyping and keeping your preliminary morphospecies in separate bags will speed up the final sorting. It can be hard to distinguish

arthropod and fungal galls. If unsure, sample all galls. Fungal galls can be identified in the laboratory and later removed from the analysis.

- Sample galled plant parts by detaching from the tree. If the galls are to be reared, and are in low numbers, galls will last longer if the plant part is attached to a twig with a couple of leaves. Otherwise, sample only the galled plant parts, preferably with active (inhabited) galls.
- Put different morphospecies in separate collecting bags. Do not overfill the bags and keep them in the shade.
- Sample enough galled material for each morphospecies to provide healthy quantities for rearing and dissection. What is considered a "healthy quantity" is dependent on the available resources (space, manpower, etc.) for rearing and dissecting, and the size of the galls. The more material reared and dissected, the better the chances of yielding insightful information to aid the species concept. Therefore, it would be ideal to rear at least 10 galled parts and retain at least 10 galls for dissection, per morphotype.
- Unsampled galls can be counted (or their abundance estimated if there are many of them; but always confirm with the expert assigning galls to final morphospecies that these galls are truly from a single morphospecies). Record the unsampled number into your notebook and report it to the sorting team after sampling.

Abundance estimates for very abundant mines and galls

Some abandoned leaf mines or gall morphospecies can be very abundant, which means counting them may take an excessive time investment. Instead of counting them individually, you can estimate their abundance in such cases. Mine and gall density can sometimes largely differ among various parts of the canopy. It is thus necessary to do the estimates repeatedly in various parts of the canopy.

- Select a reasonably large branch (ca 100-500 leaves) and count number of leaves and number of mines or galls on this branch. Divide their number by the number of leaves to calculate mine or gall per leaf average for this branch. Repeat this procedure at various parts of the canopy (at least three in the case of smaller trees and at least five in the case of larger trees). Use the averages to calculate a mean mine or gall density per individual leaf. Record this value. This can be used for estimating total mine or gall density once the total number of leaves is calculated.
- Some mite galls can be highly abundant (hundreds of galls per leaf). In such a case, pick only 20 leaves in random and calculate gall/leaf average. Repeat this procedure at various parts of the canopy (at least three in the case of smaller trees and at least five in the case of larger trees). Use the averages to calculate final mean gall density per individual leaf. Record this value. This can be used for estimating total gall density once the total number of leaves is calculated. Use this approach scarcely and only when really needed; e.g. in cases when more than 50% of leaves are galled.
- It is always better if the estimates are done by two specially trained persons using the mean estimate as a final value as it may provide more accurate results.

Spiders

Sample spiders into a vial with ethanol. All spiders from one tree can go into one vial but do not overfill it. Divide the spiders into more vials as needed to ensure a good proportion between ethanol and the sampled individuals.

Ants

Sample ants foraging on the foliage and canopy branches into a vial with ethanol. All foraging ants from one tree can go into one vial but do not overfill it. Divide the ants into more vials in such a case to ensure a good proportion between ethanol and the sample. Note that while the sampling from a crane or a cherry picker allows to do a rapid assessment of ant foragers in the canopy, it is not comparable to the ant census using felling. In the case of felling, both whole

trunk and canopy, as well as individual nests outside and inside the host tree tissue and the associated epiphytes and lianas can be sampled, measured, and distinguished from foragers (see 2.1).

3.0 Leaf area estimates and plant vouchers

Sample leaves for leaf area estimates as specified below. We estimate leaf area of mature and young leaves separately as they can harbour different herbivores. We define mature leaves as fully developed in terms of their size and thickness. Young leaves are still developing. We define young leaves as leaves which haven't reached their full size or are much softer than mature leaves. Usually, they are also more lightly coloured than mature leaves.

In addition to the leaf area estimates, use this step to obtain herbarium vouchers, which will help with confirming host-plant identification, or to measure herbivory damage. Follow standard protocols for sampling plant vouchers (e.g. Funk *et al.* 2017). Sampling plant vouchers is especially useful in areas with high tree diversity. To avoid wilting, sample vouchers in plastic bags and mark them with tags. A voucher should include a stem bearing multiple leaves and an apical bud. Always sample flowers or fruits if present. Obtain at least three vouchers from around a canopy of each tree sampled. Press and dry the vouchers on the same day they were collected. The vouchers can be later used for DNA isolation and DNA barcoding to provide additional information on species identification.

Note: Although not discussed in this study, the sampled leaves can also be used for measuring leaf physical traits and nutrient content that can be relevant for structuring insect-plant interaction networks. Sampling leaves for measuring secondary metabolites usually requires special protocols and a separate sampling campaign. For example, the samples need to be cooled or frozen immediately after the sampling to avoid degradation and oxidation.

3.1 Leaf area estimates for felled trees

1. Sample foliage for biomass estimates (Fig. P6).

i) After you have sampled the tree for arthropods, place all foliage from the canopy into bags and weigh it. For large trees (ca DBH>30 cm), you can sample 25% or 50% of the foliage and extrapolate the results if your team is small in order to speed up the process. Record the weight into the 'Plant Form'. Sample and weigh mature and young leaves separately if both young and mature leaves are present. These values will be used for separate estimates of young and mature leaf area.

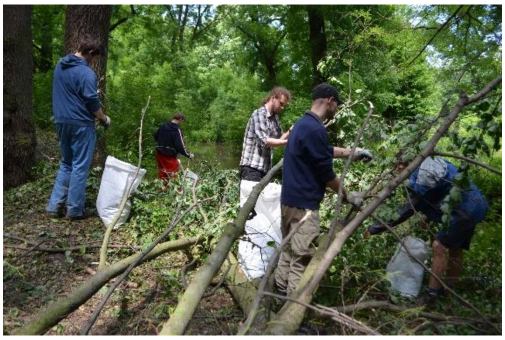
Figure P6. Leaf biomass sampling in Mikulcice.

ii) Avoid sampling leaves for biomass estimates when the foliage is wet and only sample leaves which have no other plants attached.

2. Sample leaves for calculating leaf area.

i) This includes obtaining individual leaves from across the canopy. A good method is to use the leaves sampled for the biomass estimate for this. Mix the leaves sampled for the biomass estimate in a bag and randomly pick some of them for calculating leaf area. Only use leaves which were not mechanically damaged during the sampling (but include those damaged by herbivores, pathogens, etc.).

ii) For small trees (ca. DBH < 15 cm), pick enough leaves (depending on their size) to fill a 50x50 cm white frame (Fig. P7). For larger trees or trees with large leaves, pick enough leaves to fill two frames (this is to cover the variability in

leaf sizes and shapes across the canopy of such trees). Sample young and mature leaves separately if there are both mature and young leaves present.

3. Take a photo of the leaves for the leaf area estimate.

i) Place the leaves for calculating leaf area into a 50x50 cm white frame (Fig. P7). Use as many leaves as possible but make sure they do not overlap or cross the frame border line.

ii) Leaves should be flat. Use some dark heavy objects (e.g. stones or coins) to flatten the leaves if necessary (but do not cover herbivory damage).

iii) Place a paper label with the tree number, the frame number (in case you take photos of more than one frame), and the leaf stage next to the frame so it is visible in the photo.

iv) Position the camera on a tripod right above the frame so that the frame appears on the camera display as a square.

v) Avoid strong light and shade contrasts during the photographing. Try to carry out this task with same camera settings to keep light levels consistent throughout the project.

vi) Once you take the photo, weigh the leaves. Record their total weight and their total number into the 'Plant Form'.

vii) If present, repeat this procedure for young and mature leaves separately.

viii) The resulting photos will be processed in ImageJ, Photoshop or other suitable software. In summary, the measurement is based on counting the number of pixels occupied by leaves vs. the number of pixels occupied by the background within a known area (here 2500 cm^2)). Missing leaf area or the area damaged by galls and mines can also be quantified using a similar approach in order to measure herbivory damage. Do not forget to correct for lens distortion, if needed. This can be especially important if you use a wide-angle lens. See existing protocols for details on leaf processing (e.g. Bito *et al.* 2011). The total sampled leaf area will be calculated using the total leaf biomass and the area to weight ratio from the photographed sample.

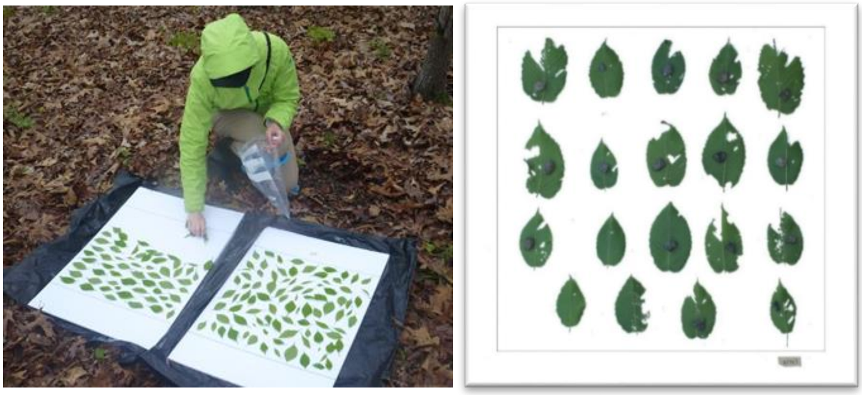

Figure P7. Preparing 50x50 cm leaf frames for photography and a final photograph of the frame. Note that the leaves are flattened by dark stones and there is a label with the tree number in the bottom corner of the frame.

3.2 Leaf area estimates for trees sampled from cranes and cherry-pickers

1. Estimate number of leaves on the tree.

i) Leaf number estimates must be done during the arthropod sampling.

ii) After you have sampled a part of the canopy for arthropods, select a reasonably large branch (with ca 500 leaves) within it and count how many leaves there are exactly (= value "A").

iii) Count how many branches of that size there are in the part of the canopy you have just sampled (= value "B"). Do this regularly. Avoid doing this across large parts of the canopy ("B" should be 5- 10, optimally).

iv) Multiply "A" with "B". Record this into your notebook as a local number of leaves ("C").

v) Repeat this procedure for each part of the canopy you sample**.**

vi) Once you finish sampling, count the sum of "C" values and report it to the ground team who will record it into 'Plant Form' as the total number of sampled leaves.

vii) Visually estimate what percentage of leaves is young and what percentage is mature if both young and mature leaves are present.

2. Sample leaves for calculating leaf area.

i) Drive the gondola all around the canopy and sample leaves in random and bring them to the ground.

ii) In the case of small trees (ca. DBH < 15 cm), pick enough leaves (depending on their size) to fill a 50x50 cm white frame (Fig. P2). In the case of larger trees or trees with large leaves, sample enough leaves to fill two frames (this is to cover variability in leaf sizes and shapes across canopy of such trees). Sample young and mature leaves separately if there are both mature and young leaves present.

3. Take a photo of the leaves for the leaf area estimate.

i) Place the leaves for calculating leaf area into a 50x50 cm white frame (Fig. P7). Use as many leaves as possible but make sure they do not overlap or cross the frame border line.

ii) Leaves should be flat. Use some dark heavy objects (e.g. stones) to flatten the leaves if necessary (but do not cover herbivory damage).

iii) Place a paper label with the tree number, the frame number (in case you take photos of more than one frame), and the leaf stage next to the frame so it would be visible on the photo.

iv) Position the camera on a tripod right above the frame so that the frame appears on the camera display as a square.

v) Avoid strong light and shade contrasts during the photographing. Try to carry out this task with same camera settings to keep light levels consistent throughout the project.

vi) Once you take the photo, weigh the leaves. Record their total weight and their total number into the 'Plant Form'.

vii) If present, repeat this procedure for young and mature leaves separately.

viii) The resulting photos will be processed in ImageJ, Photoshop or other suitable software. In summary, the measurement is based on counting the number of pixels occupied by leaves vs. the number of pixels occupied by the background within a known area (here 2500 cm^2)). Missing leaf area of the area damaged by galls and mines can be also quantified using a similar approach to measure herbivory damage. Do not forget to correct for lens distortion, if needed. This can be especially important if you use a wide-angle lens. See existing protocols for details on leaf processing (e.g. Bito *et al.* 2011). The total sampled leaf area will be calculated using the estimated total number of leaves on the tree multiplied by the mean leaf size of the photographed sample.

4.0 Sample processing and insect rearing

There can be a dedicated sorting team in the field (Fig. P8). Typically it may consist of 2-3 team members. If all team members are occupied by arthropod sampling, sample processing should be done immediately after returning from the field. The sorting team's main responsibilities are recording information into spread-sheets, sample sorting, labelling, and photographing of morphospecies and leaves.

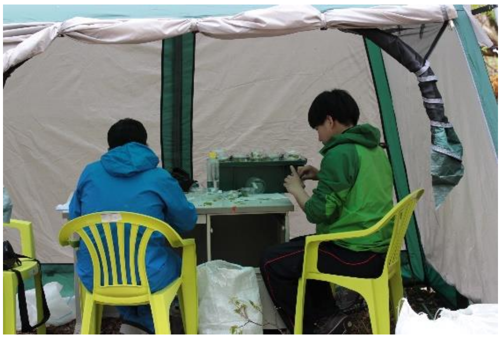
Figure P8. Sorting team in Tomakomai.

The sorting team should include team members skilled and trained in morphotyping arthropods. The initial morphotyping is done *de novo* within each individual tree. The morphospecies will be cross-referenced across all individual trees once the sampling is finished. This reduces the amount of error compared to using a system of creating morphospecies across all trees within the plot or even multiple plots. Make sure that all arthropod individuals from a given group are always morphotyped by the same person when sorting arthropods from a single tree. Minimize the number of persons involved in the morphotyping. Give this task only to the team members with a proper training. This will increase the consistency in morphotyping and lower the amount of errors.

General notes

1. Record all information about the host-plant into the 'Plant Form'.
2. Label and sort all arthropod specimens. When taking arthropod vouchers, follow available standard protocols (e.g. Millar, Uys & Urban 2000; Schauff 2001).

Leaf-chewing insect larvae

- Morphotype leaf-chewing larvae based on their morphology (e.g. size, coloration, descriptions of hairs/ spines etc.). Record morphological characteristics of each morphospecies in your notebook. It will help you to morphotype further larvae.
- A maximum of up to 50 larvae per morphospecies should be kept for rearing. Each larva is to be kept separately in a rearing container with the exception of gregarious larvae. Keep gregarious larvae from one nest together in one large zip-lock bag or container. Record the number of gregarious larvae on the label in this event.
- If there are more than 50 larvae per given morphotype (this happens rarely):
  i) Larvae 51-75 should be preserved in ethanol. Each larva should be kept in a separate vial and labelled with a standard label.
  ii) Larvae 76-x can be discarded. Fill the number of discarded larvae into the 'Plant Form'.
- Label each kept larva (use only one label per nest of gregarious larvae). Record the following information on the label:
  i) Unique Identifier (it can be pre-printed)
  ii) Locality
  iii) Tree ID number (unique number for each tree in the plot)
  iv) Morphospecies
  v) Body length (in mm)
  vi) Feeding on the host (yes/no) – to be confirmed later in the laboratory
  vii) Leaf age (record whether the larva was found on mature or young leaves)
  viii) Mode of feeding (chewing, rolling, tying, skeletizing)
  ix) Parasitized (yes/no) – to be filled in later based on the result of the rearing
  x) Reared to adult (yes/no) – to be filled in later based on the result of the rearing
  xi) Preserved in ethanol (yes/no)

- Photograph at least one larva per morphospecies. First, take a photo of the larva in detail. All important morphological characteristics (number of prolegs, setae, dorsal and lateral lines, head capsule etc.) should be visible. Take pictures from both the dorsal and lateral view (Fig. P9). Afterwards, take a photo of the same larva together with its label including all information.

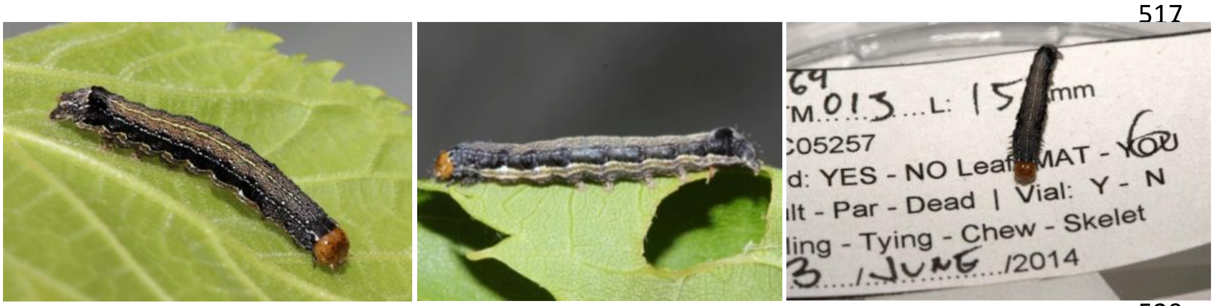

Figure P9. Morphospecies photos of a caterpillar.

Mines

- Morphotype mines based on their morphology. Record morphological characteristics of each morphospecies in your notebook. (Specifically, record whether it is a blotch or a serpentine mine, on what side of the leaf is it visible, and colour of the frass if there is any). It will help you with morphotyping future mines.
- Separate inactive mines and count them. Add this number to the number of inactive mines of the respective morphospecies reported by the sampling team and record their number into the 'Plant Form'. If you have only abandoned mines for some morphospecies, keep a mine of that morphospecies for labelling and photographing.
- Up to 50 active mines per morphospecies should be reared in zip-lock bags.
- Up to 10 other mines of the same morphospecies should be dissected. If there are less than 60 active mines in total, dissect every second mine out of first ten mines and every fifth mine of the rest. Put the dissected larvae (or any other larger remains, e.g. head capsules) in a vial with ethanol and a standard miner label.
- If there are more than 60 active mines, discard them. Add the number of mines you discarded to the number of active mines counted (but not sampled) by the sampling team (Sampling team should report this number to you). Record this number in the 'Plant Form'.
- Mines will last longer if the leaf is attached to a branch with a couple of other leaves. Do not separate them if you plan to rear them.
- Each mine is to be reared in a separate zip-lock bag. However, if there are several miners per one leaf, do not separate them. You may keep them in one zip-lock bag but put a corresponding number of labels inside.
- Label each morphospecies or larva preserved in a vial. Record following information on the label:
  i) Unique Identifier (it can be pre-printed)
  ii) Locality
  iii) Tree ID number (unique number for each tree in the plot)
  iv) Morphospecies
  v) Leaf age (record whether the mine was found on mature or young leaves)
  vi) Active/abandoned
  vii) Parasitized (yes/no) – to be filled in later based on the result of the rearing
  viii) Reared to adult (yes/no) – to be filled in later based on the result of the rearing
  ix) Preserved in ethanol (yes/no)

- Take a photo of one mine per morphospecies (Fig. P10). First, take a photo of the dorsal side in detail. Second, take a photo of the ventral side of the leaf in detail. Third, take a photo of the same mine together with its label with all information filled in and visible.

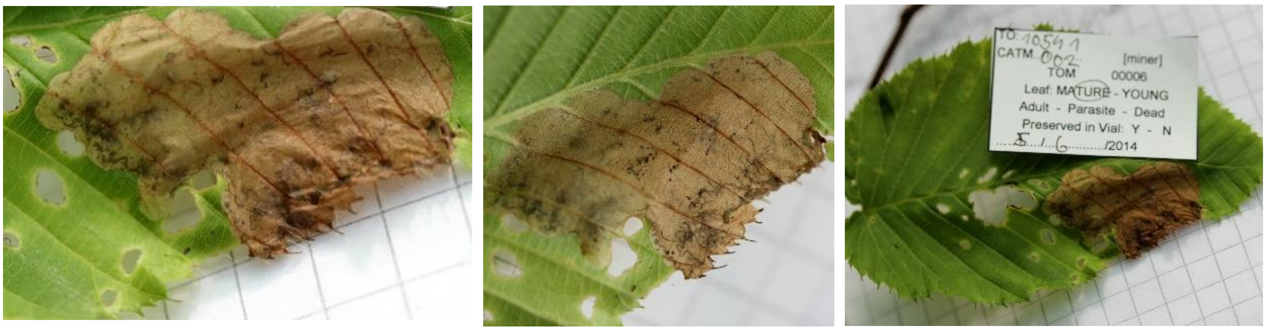

Figure P10. Morphospecies photos of a mine.

Galls

- Morphotype galls based on their morphology (mainly, record the type of the gall according to literature (e.g. Yukawa 1996; Redfern & Shirley 2002), on what side of the leaf is it visible, and its colour).
- Use the available literature and reference collections to identify fungal galls. Dissecting and examining under a microscope can be necessary for identification of fungal galls. Once you are absolutely sure about the identification, remove the fungal galls from further processing. However, if still unsure, process all galls with uncertain status. Make sure you take vouchers of such galls for further identifications by specialists.
- If galls of a morphospecies are low in number (e.g. < 15), prioritise putting them in ethanol for dissection rather than rearing.
- Select plant parts with the best looking galls (i.e. fresh, mature, no exit holes) for each morphospecies and rear them in one or more large zip-lock bags. All rearings of one morphospecies can be given the same label. Do not rear mite galls.
- Select, preferably, 10-30 individual galls per morphospecies, remove excess plant tissue, and place in ethanol for future dissection. Don't forget to add a vial label.
- Record the following information for each gall morphospecies in a separate sheet:
  i) Locality
  ii) Tree ID number (unique number for each tree in the plot)
  iii) Date
  iv) Gall morphospecies code
  v) Morphospecies description or a diagram
  vi) Plant part which was galled
  vii) Number of plant parts galled and the average number of galls per plant part. (This can be made exact if all individual galls are counted). This should also include the number of galled parts left on the tree (the sampling team should tell you if there were any). Alternatively, record the average number of galls per plant part. The number of plant parts galled can be estimated as % cover of plant parts galled (this approach is used for very abundant galls, and where the total number of tree parts will be known).
  viii) Number of galled plant parts (or individual galls) used for rearing.
- Label each morphospecies or larva preserved in a vial. Record the following information on the label:
  i) Locality
  ii) Tree ID number (unique number for each tree in the plot)
  iii) Date
  iv) Gall morphospecies code

- Take a photo of one gall per morphospecies. First, take a photo of the dorsal side in detail. Second, take a photo of the ventral side in detail. Third, take a photo of the same gall together with its label with all information filled in and visible.

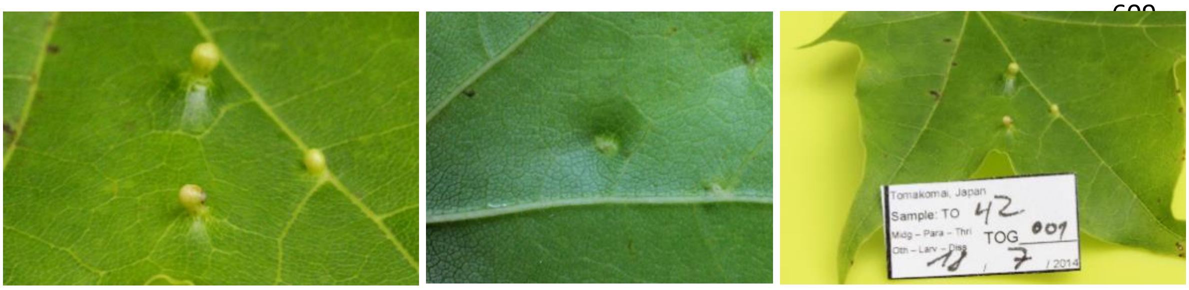

Figure P11. Morphospecies photos of galls.

Spiders

All spiders from one tree can go into one vial. Divide the spiders into more vials in the event of high spider abundance, this will ensure there is a good proportion of ethanol. Label each vial with a spider label including:

i) Locality
ii) Tree ID number (unique number for each tree in the plot)
iii) Date

Ants

When sampling from felled trees, the information on foraging ants should be directly recorded during the sampling by the person responsible (see above). In the case of sampling from cranes and cherry-pickers, the information can be recorded once the sampling of the respective tree is finished. All vials with foraging ants should be labelled with an ant label including:

Foraging ants:
i) Locality
ii) Tree ID number (unique number for each tree in the plot)
iii) Date
iv) Trunk/Canopy (record whether the ants were foraging on the trunk or in the canopy).
v) Vial number (in case there are multiple vials with foraging ants from the respective tree)

Ant nests:
Ant nests are sampled only when sampling from felled trees. We record several extra pieces of information for ant nests (such as position on the tree, nest type etc.). This information should be recorded by the responsible person directly during the sampling in 'Ant protocol' and ant labels. Once the sampling of the respective tree is finished, check whether the following information was recorded for all ant nests:
i) Locality
ii) Tree ID number (unique number for each tree in the plot)
iii) Date
iv) Position on the tree (vertical height in m from the ground)
v) Type (description of nest site, see above)
vi) Dimensions of a nest (width times height in cm, where possible to measure)
vii) Number of individuals in the nest (assessment using categorical scale of number of workers, see example of the ant protocol)
viii) Vial number (in case there are multiple nests collected from the respective tree, each nest should have its own vial)

After the tree is searched and all samples collected, make sure that all the vials have the proper information written on their labels, and that all information is also described in the ant protocol for each tree (and that both the protocol, and labels match). Make sure all vials are full of ethanol. Check that vials are well closed/not leaking!

Take a photo of each different nest type for the common ant species, or their association with plant/symbiont species (see below). It is not necessary to take photos of all nests, but all common cases should be documented at least 3 times. The photograph should include the nest label (tree number + vial number), the voucher itself, and a scaler in cm.

Optional additions to the ant protocol:

Although not discussed in this study, the protocol for sampling ant nests can be also used for sampling other arthropods. Apart from ants, this protocol can be used for sampling termites, and the ant/termite associated trophobionts and symbionts (aphids, scale insects, beetles, bugs etc.). If the ant protocol is extended in this way, the same procedure is followed. In this case, mark if the sample contains ants, termites, or symbionts in the protocol (see example of the protocol (*"Ant, Ter, Sym"* marks) and examples of the labels). A small sample of ant individuals (1-5 workers) should be always collected with the symbionts to confirm host associations.

Insect rearing

All sampled larval insect herbivores should be reared to adults or parasitoids. Always protect rearing containers and bags from direct sunlight. Appropriate temperature and humidity are key factors affecting the rearing success. Always keep your rearing containers clean. Check them frequently and remove any frass or other waste to prevent growth of fungi. When taking vouchers of the reared arthropods, follow standard protocols (e.g. Millar *et al.* 2000; Schauff 2001), unless otherwise specified (see below).

Leaf-chewers

- Leaf-chewers should be reared in either plastic containers or zip-lock bags for large nests of gregarious larvae (Fig. P12). Write the most important information (host tree individual, morphotype number) on the container. This will serve as a back-up source of the most important information if the label gets mouldy or eaten by the larva.
- Inspect the containers every day.
- Provide larvae with fresh leaves and clean the boxes if necessary. This is usually needed every second day at least.
- Put some tissue paper into the bags or containers to absorb condensed water if needed.
- Record whether the larva feeds on mature or young leaves (mark it in the label). Record the mode of feeding if it hasn't been recorded already.
- Once the larvae pupate, clean the container. Remove any remaining old leaves, unless the pupa is directly attached to them. If this occurs, remove as much of the leaf tissue as possible without damaging the pupa. This will reduce the risk of fungal infection. Put some paper tissue or toilet paper inside the containers. This can either be used to absorb extra moisture (if you rear the pupae in a humid environment) or can be moistened if you rear the pupae in an environment with low air humidity. Separate the pupated individuals from the active larvae and check the container every day.
- Record if the larva died or was reared to an adult or a parasitoid. If it died, mark whether it was preserved in a vial with ethanol or not.
- Kill and mount every reared Lepidoptera adult. Killing by freezing will assure the best quality of DNA for barcoding. Abundant species with a known identification can be just pinned. Store adults in a dryer overnight. Place them in storage boxes once they are dry.

- Store reared parasitoids in ethanol. Label them with all of the host information as well as a unique parasitoid code.
- Note that many temperate insect species overwinter as pupae and you won't be able to rear their larvae into adults within a single season. Plan your project accordingly.

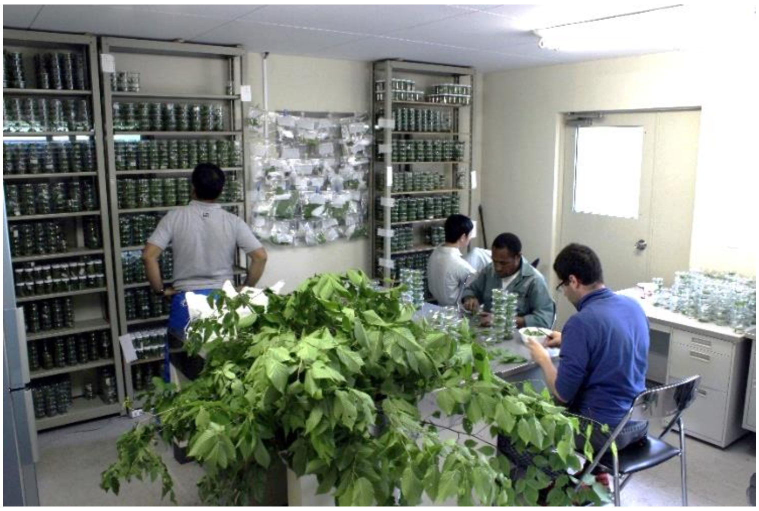

Figure P12. Insect rearing in Tomakomai. Note the caterpillars being reared in plastic containers placed in the shelves. Gallers and miners are reared in plastic bags hanging on the wall. All larvae are checked regularly

Mines and galls

- Mines and galls are reared in plastic bags (Fig. P12). Inspect the bags every day.
- Put some paper tissue or toilet paper into the bags to absorb condensed water.
- Record if the larva died or was reared to an adult or a parasitoid. If it died, mark whether it was preserved in a vial with ethanol or not.
- Kill and immediately mount every reared Lepidoptera adult. Store adults in a dryer overnight. Place them in storage boxes once they are dry. Mining and galling Microlepidoptera may die relatively quickly after emerging. It is thus essential to check for emerging adults regularly, ideally twice a day.
- Once dead, Microlepidoptera adults dry quickly due to their small size and are hard to relax for mounting. Therefore, if they die spontaneously in the rearing bag or container they are very difficult to mount. Store such individuals dried and fixed in Eppendorf tubes (but try to avoid such a situation in general!).
- Importantly, mounting mining and galling Microlepidoptera adults requires training. Study and follow standard protocols on Microlepidoptera mounting (e.g. Landry & Landry 1994).
- Adult Hymenoptera, Diptera, and Coeloptera should be preserved in vials with ethanol.
- Store reared parasitoids in ethanol. Do not forget to add a label with all information on the original herbivore larva.
- Mines and galls which do not emerge in 30 days can usually be discarded in tropical areas. If you are working in temperate regions, inform yourself if there are any owewintering species associated with your focal host plants. Such species should be kept over winter. In addition, dissect a representative number of mines and galls per morhospecies before discarding. If there are any macroscopic remains of the larvae (e.g. head capsulas), preserve them in a vial and ethanol with a standard label.

Rearing rare mine or gall morphospecies

In the case of rare morphospecies of galls and mines, which were sampled as a single leaf (without sufficient other plant parts attached) follow the rearing protocol by Ohshima (2005):

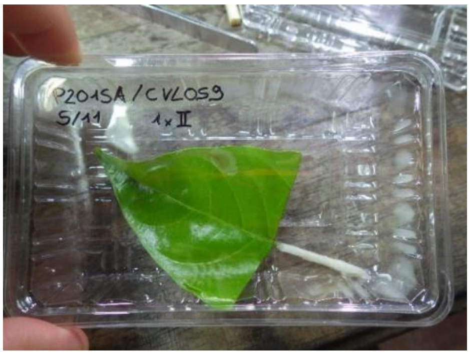

- Remove the basal part of the leaf and expose the central vein.
- Prepare 1% sucrose solution and dip a piece of clean wiping paper in it.
- Wrap the petiole and exposed part of the central vein with the wiping paper.
- Store the leaf in a plastic container
- Check the container twice a day.
- Replace the wiping paper regularly (usually in two day intervals).

References

Bito, D., Novotny, V., Burwell, C., Nakamura, A., Kitching, R. & ØDEGAARD, F. (2011) Predator pressure, herbivore abundance and plant damage along a subtropical altitudinal gradient. *Memoirs of the Queensland Museum,* **55**.

Funk, V.A., Gostel, M., Devine, A., Kelloff, C.L., Wurdack, K., Tuccinardi, C., . . . Coddington, J. (2017) Guidelines for collecting vouchers and tissues intended for genomic work (Smithsonian Institution): Botany Best Practices. *Biodiversity Data Journal*.

Landry, J.-F. & Landry, B. (1994) A technique for setting and mounting microlepidoptera. *J. Lepid. Soc,* **48,** 2.

Millar, I., Uys, V. & Urban, R. (2000) *Collecting and preserving insects and arachnids*. ARC - Plant Research Institute, Pretoria.

Ohshima, I. (2005) Techniques for continuous rearing and assessing host preference of a multivoltine leaf-mining moth, Acrocercops transecta (Lepidoptera: Gracillariidae). *Entomological Science,* **8,** 227-228.

Redfern, M. & Shirley, P. (2002) *British Plant Galls: Identification of Galls on Plants and Fungi,* Second edition edn. FSC Publications, Shrewsbury.

Schauff, M.E. (2001) *Collecting and preserving insects and mites: techniques and tools*. Systematic Entomology Laboratory, USDA, Washington DC.

Yukawa, J. (1996) *Insect and mite galls of Japan in colors*. Zenkoku Noson Kyoiku Kokai.

Example 'Plant Form' (felling)

TREE ID nr.: _______ SPECIES:_______________

CUT DOWN DATE: ____ __________ **RECORDED BY:** ________________

PLANT SIZE: DBH __________ HEIGHT _________

NUMBER OF PLANT VOUCHERS TAKEN:

TRUNK HEIGHT ______________________________ Meters
[from the ground to the first big branches]
CROWN HEIGHT ______________________________ Meters
[from the first big branches to the top]
CROWN WIDTH ______________________________ Meters
[across the branches]
LEAVES: MATURE/YOUNG

PERCENTAGE OF THE FOLIAGE SAMPLED:

MATURE LEAVES:

WEIGHT of the foliage _________________ **KG**

% of the foliage sampled for biomass estimate:

LEAVES FRAME WEIGHT _____________ **GRAMS**

LEAVES FRAME - leaf area:____ __________ cm^2

NUMBER OF LEAVES IN FRAME:_ ______

Leaf area before herbivory ___________ cm^2 Leaf area after herbivory ___ _______ cm^2

No. of discs _______ diameter: ______________

YOUNG LEAVES:

WEIGHT of the foliage _________________ **KG**

% of the foliage sampled for biomass estimate:

LEAVES FRAME WEIGHT _____________ **GRAMS**

LEAVES FRAME - leaf area:____ __________ cm^2

NUMBER OF LEAVES IN FRAME:_ ______

Leaf area before herbivory ___________ cm^2 Leaf area after herbivory ___ _______ cm^2

No. of discs _______ diameter: ______________

NUMBER OF ABANDONED MINES (can be a real number or % of the foliage attacked):

CAT001	CAT002	CAT003	CAT004	CAT005	CAT006	CAT007	CAT008	CAT009	CAT010

NUMBER OF DISCARDED ACTIVE MINES (can be a real number or % of the foliage attacked):

CAT001	CAT002	CAT003	CAT004	CAT005	CAT006	CAT007	CAT008	CAT009	CAT010

NUMBER OF DISCARDED CATERPILLARS:

CAT001	CAT002	CAT003	CAT004	CAT005	CAT006	CAT007	CAT008	CAT009	CAT010

CAT011	CAT012	CAT013	CAT014	CAT015	CAT016	CAT017	CAT018	CAT019	CAT020

NOTE:

Example 'Plant Form' (canopy cranes and cherry-pickers)

TREE ID nr.:________ SPECIES:________________

SAMPLING DATE: ____ __________ 2016 RECORDED BY: _________________

PLANT SIZE: DBH __________ HEIGHT _________

NUMBER OF PLANT VOUCHERS TAKEN:

TRUNK HEIGHT Meters
[from the ground to the first big branches]

CROWN HEIGHT Meters
[from the first big branches to the top]

CROWN WIDTH Meters
[across the branches]

LEAVES: MATURE/YOUNG

PERCENTAGE OF THE FOLIAGE SAMPLED:

ESTIMATED NUMBER OF MATURE LEAVES:

MATURE LEAVES FRAME - leaf area:____ cm^2

NUMBER OF MATURE LEAVES IN FRAME:_ ______

Leaf area before herbivory ___cm^2 Leaf area after herbivory ___ cm^2

No. of discs ________ diameter: ________________

ESTIMATED NUMBER OF YOUNG LEAVES:

YOUNG LEAVES FRAME - leaf area:____ cm^2

NUMBER OF YOUNG LEAVES IN FRAME:_ ______

Leaf area before herbivory ___cm^2 Leaf area after herbivory ___ cm^2

No. of discs ________ diameter: ________________

NUMBER OF ABANDONED MINES:

CAT001	CAT002	CAT003	CAT004	CAT005	CAT006	CAT007	CAT008	CAT009	CAT010

NUMBER OF DISCARDED ACTIVE MINES:

CAT001	CAT002	CAT003	CAT004	CAT005	CAT006	CAT007	CAT008	CAT009	CAT010

NUMBER OF DISCARDED CATERPILLARS:

CAT001	CAT002	CAT003	CAT004	CAT005	CAT006	CAT007	CAT008	CAT009	CAT010

CAT011	CAT012	CAT013	CAT014	CAT015	CAT016	CAT017	CAT018	CAT019	CAT020

NOTE:

Example 'Ant Protocol'

Tree No:		No. of Vials:	
Date: 2018		Collector:	

Myrmecophyte: YES / NO (Domatia/Nectaries) No.: vial number. Ant - ants, Ter - termites, Sym - symbionts

T = collection on the trunk bellow branches, C = collection in the crown; N = nest, F = only foraging ants/termites

No		Nest characteristic and notes		Nest location
1	Ant-Ter Sym T - C N - F			Distance from the ground:
				……, … m
		Is whole colony collected?	Estimation of workers number (for a nest)	Size of colony w x h
		YES / NO	<100 100-500 501-1000 >1000	…… x …… cm
2	Ant-Ter Sym T - C N - F			Distance from the ground:
				……, … m
		Is whole colony collected?	Estimation of workers number (for a nest)	Size of colony w x h
		YES / NO	<100 100-500 501-1000 >1000	…… x …… cm
3	Ant-Ter Sym T - C N - F			Distance from the ground:
				……, … m
		Is whole colony collected?	Estimation of workers number (for a nest)	Size of colony w x h
		YES / NO	<100 100-500 501-1000 >1000	…… x …… cm
4	Ant-Ter Sym T - C N - F			Distance from the ground:
				……, … m
		Is whole colony collected?	Estimation of workers number (for a nest)	Size of colony w x h
		YES / NO	<100 100-500 501-1000 >1000	…… x …… cm
5	Ant-Ter Sym T - C N - F			Distance from the ground:
				……, … m
		Is whole colony collected?	Estimation of workers number (for a nest)	Size of colony w x h
		YES / NO	<100 100-500 501-1000 >1000	…… x …… cm
6	Ant-Ter Sym T - C N - F			Distance from the ground:
				……, … m
		Is whole colony collected?	Estimation of workers number (for a nest)	Size of colony w x h
		YES / NO	<100 100-500 501-1000 >1000	…… x …… cm
7	Ant-Ter Sym T - C N - F			Distance from the ground:
				……, … m
		Is whole colony collected?	Estimation of workers number (for a nest)	Size of colony w x h
		YES / NO	<100 100-500 501-1000 >1000	…… x …… cm
8	Ant-Ter Sym T - C N - F			Distance from the ground:
				……, … m
		Is whole colony collected?	Estimation of workers number (for a nest)	Size of colony w x h
		YES / NO	<100 100-500 501-1000 >1000	…… x …… cm
9	Ant-Ter Sym T - C N - F			Distance from the ground:
				……, … m
		Is whole colony collected?	Estimation of workers number (for a nest)	Size of colony w x h
		YES / NO	<100 100-500 501-1000 >1000	…… x …… cm

Example 'Ant Labels' and how to fill them. For examples of herbivore labels, see Figures P9-P11.

Frontal side of the label:	Back side - hand notes examples:
PNG, Madang prov.Wannang vill.<br>Tree: W S -- 1A -- 1000<br>T/C # 1 | 22 Apr 2018<br>Height .... m Whole col. Y / N	Ant foraging on crown
PNG, Madang prov.Wannang vill.<br>Tree: W S -- 1A -- 1000<br>T /C # 2 | 22 Apr 2018<br>Height 12 m Whole col. Y / N	ant nest in soil under epihyte roots
PNG, Madang prov.Wannang vill.<br>Tree: W S -- 1A -- 1000<br>T /C # 3 | 22 Apr 2018<br>Height 10 m Whole col. Y / N	termite carton nest on trunk
PNG, Madang prov.Wannang vill.<br>Tree: W S -- 1A -- 1000<br>T/C # 4 | 2 Apr 2018<br>Height 22 m Whole col. Y / N	ant nest in live hollow twig
PNG, Madang prov.Wannang vill.<br>Tree: W S -- 1A -- 1000<br>T/C # 5 | 2 Apr 2018<br>Height 22 m Whole col. Y / N	symbionts of nest #4 inside live twig
